# Veterinary and pet owner perspectives on addressing access to veterinary care and workforce challenges

**DOI:** 10.3389/fvets.2024.1419295

**Published:** 2024-07-04

**Authors:** Rebecca Niemiec, Veronica Champine, Danielle Frey, Allyce Lobdell, Apryl Steele, Claire Vaiden, Lori Kogan, Andrew Mertens

**Affiliations:** ^1^Animal-Human Policy Center, Human Dimensions of Natural Resources Department, Colorado State University, Fort Collins, CO, United States; ^2^College of Veterinary Medicine and Biomedical Sciences, Colorado State University, Fort Collins, CO, United States; ^3^Dumb Friends League, Denver, CO, United States; ^4^Bureau of Animal Protection, Colorado Department of Agriculture, Broomfield, CO, United States; ^5^School of Public Health, University of California, Berkeley, Berkeley, CA, United States

**Keywords:** access to care, workforce, telehealth, scope of practice, veterinary professional associate

## Abstract

**Objectives:**

(1) Assess and compare the perceptions of pet owners and veterinary professionals pertaining to the extent of veterinary workforce and access to care challenges in 2023 in Colorado, and (2) Assess what programs, policies, and resources veterinary professionals and pet owners believe would be most effective at addressing access to care and workforce challenges in Colorado.

**Sample:**

736 veterinarians, veterinary technicians, or practice/owner manager (“veterinary professionals”) in Colorado. A total of 1,209 pet owners (919 from an online survey and 290 from in-person surveying).

**Procedures:**

Distribution of an online anonymous survey to veterinary professionals in Colorado. Pet owners were surveyed both online and in-person at pet pantry or shelter events.

**Results:**

Veterinary professionals reported significant workforce challenges, including having to frequently divert clients, clients forced to decline medical care or having to euthanize their pets due to cost. Veterinary professionals were especially supportive of policy efforts to enhance recruitment and retention of technicians, including through mechanisms such as clarifying their scope of practice, loan repayment programs, and enhancing career pathways. Colorado pet owners’ responses pertaining to the scope of access to care challenges were similar to prior national research. Pet owners reported particularly needing low-cost emergency clinics in their community as well as resources to reduce the cost of care. Pet owners were generally supportive of expanding veterinary care access through telemedicine; indicating they would feel comfortable seeing a veterinarian via telemedicine, even for the first time, and that expanded use of telemedicine would increase their ability to obtain care.

**Conclusions and clinical relevance:**

Colorado pet owners and veterinary professionals both identified numerous access to care challenges as well as indicated support for the development of several potential initiatives to address the problem. Low-cost clinics that provide sick and emergency care was the resource rated as being most helpful among pet owners. Further exploration of grants, voucher programs, expansion of telemedicine, increased utilization and title protection for CVTs, and the creation of the veterinary professional associate position are all initiatives that were noted to be worthy of further exploration.

## 1 Introduction

National studies have found that more than 25% of US pet owners struggle to access veterinary care (1, 2). Between 2017–2018, 21% of dogs and 52% of cats in the US had not received any routine or preventative care [AVMA, (3) Pet Ownership and Demographic Sourcebook]. Lack of access to veterinary care not only poses a significant threat to animal health, but also to human well-being (2). Approximately 95% of pet owners in the US consider their pets to be members of the family (4) and enjoy a myriad of physical and emotional benefits from their pets including increased physical activity, reduced cardiovascular risk, and decreased social isolation (5–10). Lack of access to veterinary care, therefore, poses a threat to bonded families and the benefits that people in these families receive from the human-animal bond (2, 8, 11, 12).

The rising cost of veterinary care has been identified as a primary factor preventing owners from accessing veterinary care (1). Between the 1990’s and the 2010’s, the cost of veterinary medical care rose faster than both human health care and inflation, with a corresponding increase in the number of pets not receiving care (13, 14). Additional barriers preventing pet owners from obtaining veterinary care include geographical distance to a veterinary clinic, lack of transportation, lack of trust in veterinarians, owners’ beliefs that they can provide care themselves, and language barriers (1, 15, 16). One study, for example, found that only 8% of US veterinary clinics have Spanish-speaking staff (17).

A growing body of literature has also highlighted the challenges facing veterinary professionals in their efforts to provide care to all patients (18, 19). In a survey of over 1,000 veterinarians in the United States and Canada, Kipperman et al. (19) found that the majority of respondents reported that their clients’ economic limitations affected their ability to provide the desired care for their patients on a daily basis. Many veterinary professionals have reported that client economic limitations, as well as high caseloads, are important contributing factors to mental health and workplace burnout (19, 20) and in some circumstances, their decisions to leave clinical practice (21).

A myriad of solutions have been offered to address these workforce and access to care challenges, some of which have been implemented through community or state-wide policies and programs. Community-based programs have been implemented that seek to reduce cost, transportation, and other barriers to care through interventions such as mobile or low-price spay/neuter clinics, angel funds, vouchers for discounted veterinary care for income qualified pet owners, and low-price full-service clinics (15). Relatedly, programs have been proposed which connect pet families in need with veterinary service providers, community groups, and social service agencies and help cover the costs of care [e.g., AlignCare, (2)]. On a policy-level scale, efforts to address access to care and workforce challenges have typically focused on enhancing recruitment and retention of veterinary professionals (e.g., through loan repayment programs; credentialing, providing title protection, clarifying and expanding the role of credentialed technicians, or the introduction of the veterinary professional associate position), encouraging professionals to provide a spectrum of care options that meet the economic needs of clients, and enhancing access to telemedicine (22–25).

Despite the diversity of solutions being discussed, little research has examined what pet owners and veterinary professionals believe would help address the access to care and veterinary workforce challenges in their community. Rather, existing research has focused primarily on understanding the scope of these problems and what is currently being done to address these issues by veterinary professionals, shelters, animal welfare organizations, and others (15). When studies have examined veterinary professionals’ perspectives on potential policies, they have typically focused on one potential policy solution, such as the veterinary professional associate proposal, which limits the ability to conduct comparisons of support across policies and programs (26, 27). Hearing directly from the populations affected by access to care and workforce challenges on what they would like to see implemented to address these challenges is essential to developing well-informed policies and programs.

To address these gaps in knowledge, we surveyed pet owners and veterinary professionals in Colorado to understand their perspectives on potential programs, policies, and resources to address veterinary workforce and access to care challenges in the state. We examined both pet owners’ and veterinary professionals’ experiences so that we could understand the potential challenges from the demand and supply side of the issue and triangulate what might be the most well-supported solutions. We also examined the extent of veterinary workforce and access to care challenges, including barriers to accessing care, frequency of turning away clients and finance driven euthanasia, and mental health impacts of these challenges, to understand whether Colorado is similar to the broader US in the problems facing pet owners and veterinarians.

Our study sought to answer the following two research questions:

1. What are the perceptions of pet owners and veterinary professionals pertaining to the extent of veterinary workforce and access to care challenges in Colorado?
2. What programs, policies, and resources do veterinary professionals and pet owners believe would be most effective at addressing access to care and workforce challenges in Colorado and why?

## 2 Materials and methods

Our research team used a collaborative process to design and distribute the surveys with a task force that included key leaders from industry, academia, non-profit organizations, and government working on access to care and veterinary workforce challenges in Colorado. The iterative process involved a collaborative literature review, meetings to build consensus on key topics to include in the surveys, and opportunities for all taskforce members to provide feedback on multiple survey drafts. Based on this process, both surveys were designed to first include a series of questions seeking to understand respondents’ perspectives on and experiences with workforce and access to care challenges (Research Question 1).

After exploring the scope of the challenges, both surveys were then designed to have a second section focused on potential policies, resources, or programs seeking to address these challenges (Research Question 2). In the veterinary professional survey, this section asked about respondents’ perspectives on the following potential programs and policy solutions that were being discussed in Colorado and in other states: (1) clarifying the roles of registered/certified veterinary technicians (hereby referred to as “CVT’s”) and advancing career pathways for said technicians; (2) implementation of grants, vouchers, or programs like AlignCare to expand access to care (2); (3) loan repayment programs and educational assistance; (4) the introduction of a veterinary professional associate; and (5) expanding the use of telemedicine.

In this second section on solutions, the pet owner survey was designed to focus more on programs and resources for expanding access to care, rather than workforce solutions. The pet owner survey therefore asked respondents to rate 18 different programs, policies, or resources that the taskforce identified as being currently discussed or implemented in various US states or regions to increase access to care. To complement the veterinary professional survey data, the pet owner survey also asked respondents’ their comfort in having their pet seen for medical care by various veterinary professionals for different procedures and their comfort in using telemedicine. Both surveys asked a series of demographic and respondent specific data to enable our research team to analyze results by subgroups. Surveys were pre-tested by members of the task force, who piloted both surveys themselves and shared the surveys with those in their network for pre-testing. Pre-testing led to several changes in language in the questions to enhance clarity. See Supplementary materials for the final copies of the surveys.

### 2.1 Recruitment of survey participants

Recruitment for the veterinary professional survey occurred in August 2023 via a combination of mailed postcards and email listservs. The research team sent survey recruitment postcards to all 5,758 veterinary professionals on the Colorado Department of Regulatory Agencies (DORA) list of licensed veterinarians and veterinary technicians. The postcard included a QR code and link to the online survey. A follow up postcard was sent out several weeks later to all individuals on the DORA listserv. A link to the online survey was also included in newsletters sent out by the Colorado Veterinary Medical Association (CVMA) and the Colorado Association for Certified Veterinary Technicians (CACVT). Taskforce members also sent the survey out via email to networks of Colorado veterinary professionals they were aware of (e.g., email networks of practice owners/managers). In all communications, it was requested that the survey not be posted on social media to ensure that the survey was not available to the general public and was only distributed to professionals. The first question of the survey asked respondents if they were currently a veterinarian, veterinary technician, or practice owner/manager in Colorado. If they selected “No,” the survey was terminated.

In this manuscript, for simplicity, we use the phrase “certified veterinary technicians” (CVTs) when referring to technicians; however the survey used certified/registered veterinary technicians (CVTs/RVTs) throughout because veterinary technicians in Colorado became regulated by the State Board of Veterinary Medicine beginning January 2023 and must be registered according to Part 2 of the Veterinary Practice Act. Throughout 2023, certified veterinary technicians (CVTs) will be transitioning to registered veterinary technicians (RVTs), so both titles were included.

Two different samples for the pet owner survey were recruited: (1) a sample of pet owners who are representative of the broader population in Colorado in terms of demographics, recruited through the online panel provider Qualtrics (Provo, UT) and (2) a sample of pet owners, recruited in person, who might be experiencing barriers to access to veterinary care. The representative Qualtrics sample was recruited to allow us to estimate the scope and type of challenges that pet owners throughout the state are experiencing, while the second sample was recruited to ensure we obtained the perspectives of those with the most potential need for programs and policies focused on access to care.

The representative pet owner sample, recruited via the online panel provider Qualtrics, were incentivized to participate in different ways, depending on which panel they were recruited from (e.g., monetary incentives, game points, gift cards, or other prizes). We adopted a stratified sampling approach to ensure that the sample was representative of the overall Colorado public in terms of age, gender, and income [as reported by the US Census Bureau American Community Survey (28)] and had a target sample size of 200 participants for each of the three regions (Front Range, Eastern Plains and Western Slope) in Colorado.

To obtain a sample of pet owners who might be experiencing the most barriers to care, we collaborated with the Colorado Pet Pantry, Annie and Millie’s, and the Roice-Hurst Humane Society to survey participants at 11 events which were held throughout the state in September and October 2023. Ten of the 11 events were pet food pantry events, while one was a low cost vaccine clinic at a shelter. At these events, all attendees were approached by a member of our research team and asked if they would complete a shorter paper version of the survey that included questions about access to care challenges and ratings of 18 potential programs but excluded questions about telemedicine and comfort with different veterinary professional scopes of work. Surveys were available in English and Spanish. For both pet owner surveys, respondents were asked if they currently had a dog or cat or had a dog or cat in the past two years; if they selected “No,” their survey was terminated and their response was not included.

### 2.2 Data management and analysis

Both online surveys (i.e., the veterinary professional and representative public survey) were conducted using Qualtrics software, and results were downloaded from this software into CSV files kept in password protected folders only available to the research team. The results of the in-person survey were digitized by hand by the research team, by recording responses into a CSV file to mirror how the Qualtrics results were presented. Descriptive analyses (e.g., means, frequencies) were tabulated using the R programming language. We did not conduct any hypothesis testing as our research questions were exploratory and focused on overall perceptions and support; therefore, we primarily report descriptive statistics below.

## 3 Results

### 3.1 Descriptions of samples

For our veterinary professional survey, we received a total of 775 initial responses; of these, 736 responded that they agree to terms of the survey and that they are a veterinarian, veterinary technician, or practice/owner manager in Colorado. Given that we sent a total of 5,758 postcards in the mail to veterinary professionals, our response rate was 12.7%. Of these respondents 415 (57%) received the survey via the postcard mailing, 114 (15%) were forwarded the survey from a friend or colleague, 151 (20.5%) received it from an email listserv, 27 (3.6%) received it via social media, and 52 (7.1%) received it via “other” source. A total of 418 respondents completed 100% of the survey while 493 completed at least 60% of the survey. We included all partial (i.e., incomplete) responses in our analyses and did not have a minimum completeness rate. When reporting our results, we provide the percentage of respondents who provided a particular answer to each question over the total number of respondents who answered the question (which varies by question).

**Figure 1:**
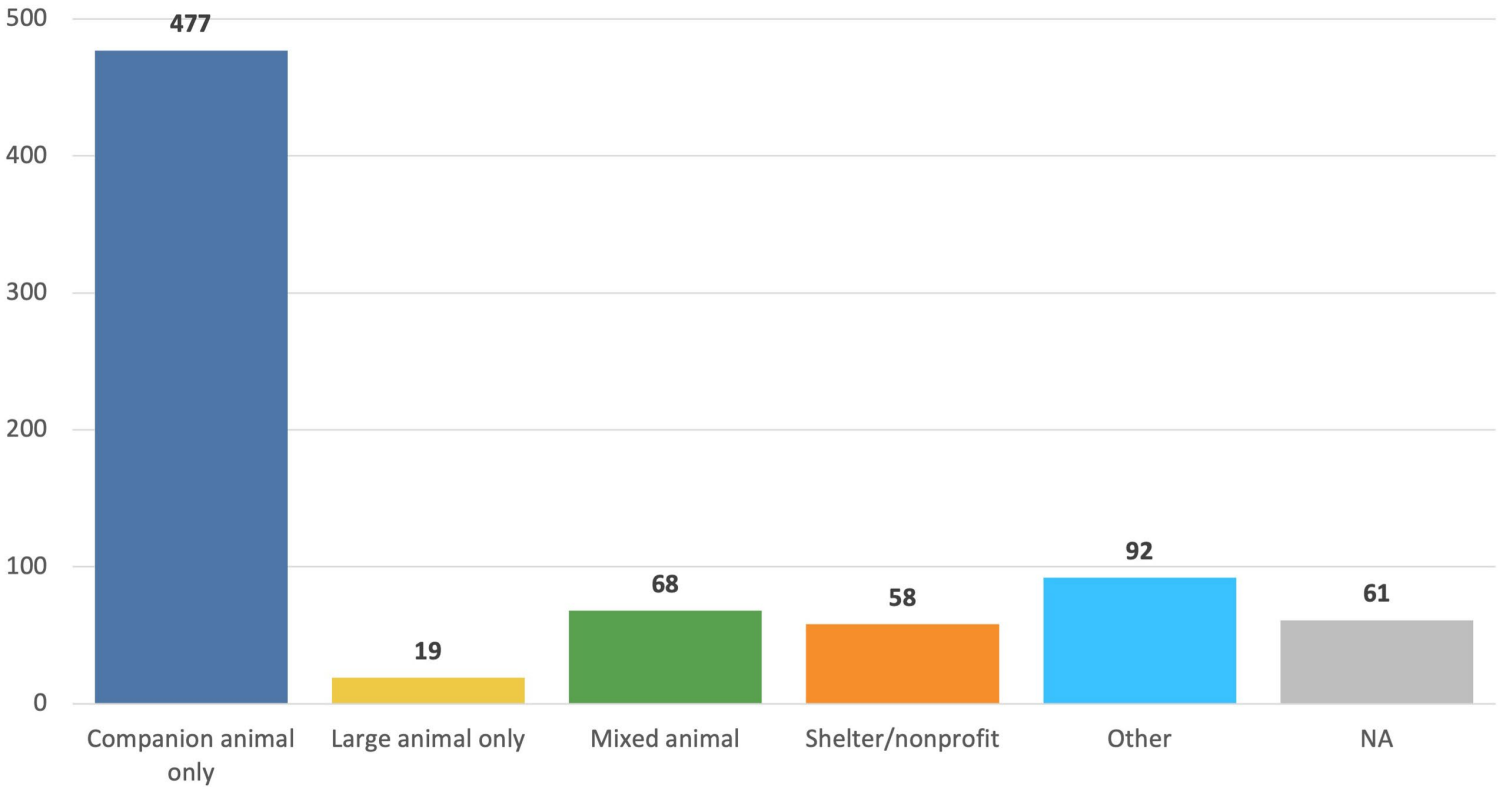
The total number of veterinary professionals (out of *n* = 736) who completed the survey who work in companion animal, mixed, large animal, shelter or other types of practices.

The majority (*n* = 477,65%) of respondents worked in companion animal only practices (Figure 1). A total of 287 (39%) reported that they worked for a privately owned practice, 255 (35%) reported that they worked for a corporately owned practice, and 18 reported that they worked for a non-profit (2.4%). A total of 269 (37%) of the respondents reported that their current role was a veterinarian, 385 (52%) reported that their role was a registered/certified veterinary technician or non-credentialed veterinary technician, 126 (17%) reported being a practice owner/manager, and 30 (4%) reported “other.” A total of 49 (7%) of all respondents indicated they were both practice managers/owners and DVMs. Of the DVMs, 75 (35%) worked for a corporately owned practice, while 129 (61%) worked for a privately owned practice. Of the practice managers/owners, 20 (36%) worked for a corporately owned practice while 33 (59%) worked for a privately owned practice.

For our pet owner surveys, we received a total of 919 responses from the Qualtrics panel. The demographics of the sample mostly mirrored the census estimates (Table 1), except that the sample had slightly fewer younger respondents and slightly more respondents with income less than $50,000. A total of 494/919 (53.8%) of respondents currently owned or regularly cared for a cat, while 730/918 (79.5%) currently owned a dog. A total of 305/918 (33.2%) had owned or regularly cared for a cat in the past two years that they no longer had, and 443/919 (48.2%) had owned a dog in the past two years that they no longer had. There were 50/919 or 5.4% of respondents in the online sample who had owned a dog/cat in the past two years but did not currently have a dog/cat. We received a total of 290 responses from in-person surveying of pet owners at pet pantry or shelter events. A total of 161/282 (57.1%) of respondents currently owned or regularly cared for a cat, 245/283 (86.6%) currently owned a dog, 104/276 (37.7%) had owned or regularly cared for a cat in the past two years that they no longer had, and 151/274 (55.1%) had owned a dog in the past two years that they no longer had. All respondents currently owned a dog or cat. A total of 261 (90.0%) of the surveys were completed in English, while 29 (10.0%) were completed in Spanish.

**Table 1:** The demographics of the 919 pet owners surveyed in the Qualtrics online panel compared to the 2022 American Community Survey Census estimates for those variables.

Demographic	Online sample	Census ESTIMATES
**Gender**		
Man	431/914 (47.2%)	51%
Woman	478/914 (52.3%)	49%
Non binary	5/914 (0.5%)	<1%
**Age**		
18–34	263/919 (28.6%)	32%
35–54	327/919 (35.6%)	34%
55+	329/919 (35.8%)	35%
**Household income**		
Less than $50,000	296/919 (32.2%)	27%
$50,000–$100,000	282/919 (30.7%)	29%
Over $100,000	341/919 (37.1%)	44%

### 3.2 Veterinary professional survey—workforce challenges related to serving clients reported

**Figure 2:**
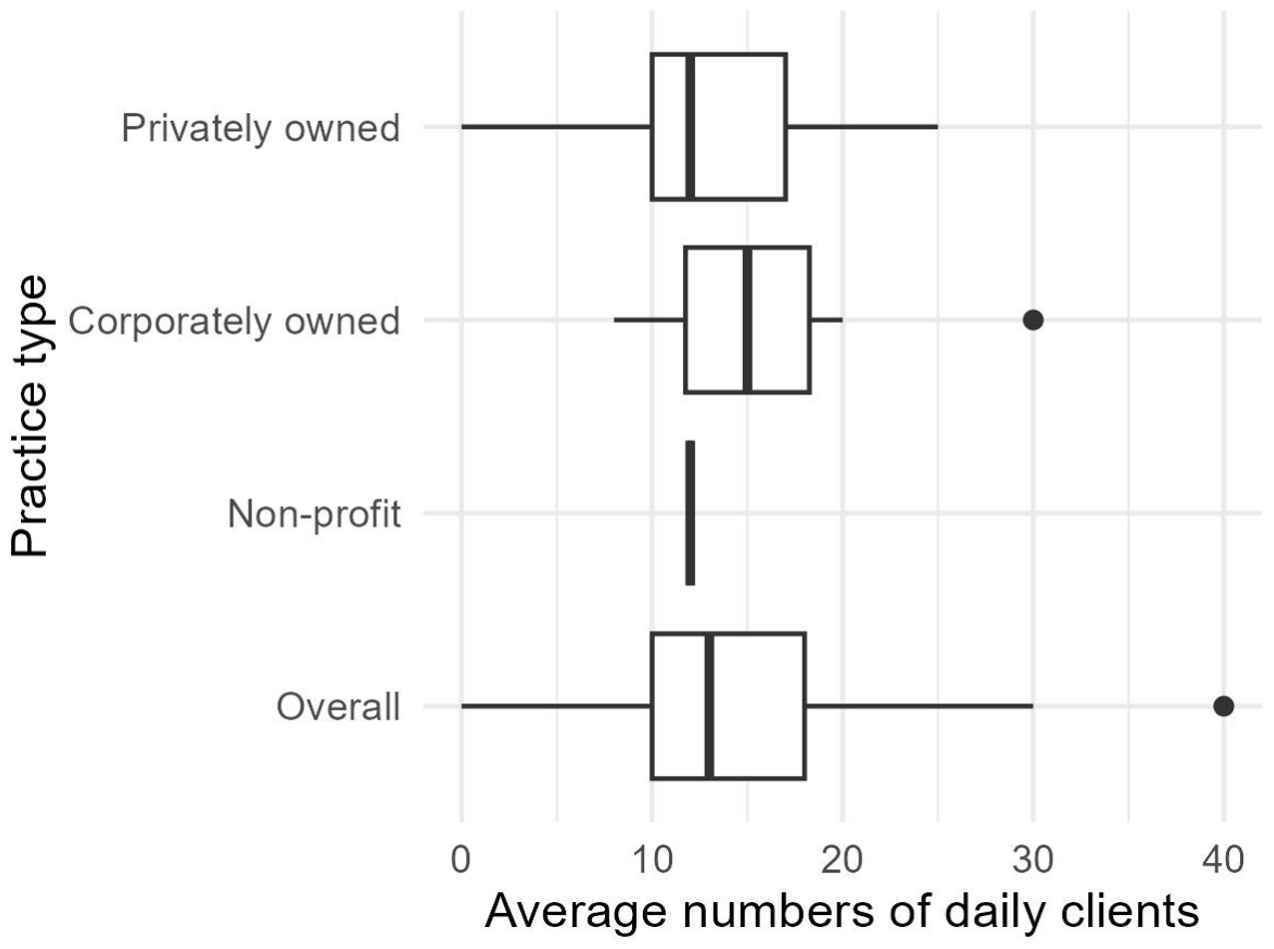
Responses to the question, “On average, how many clients does your practice schedule per veterinarian per day,” by type of practice.

**Figure 3:**
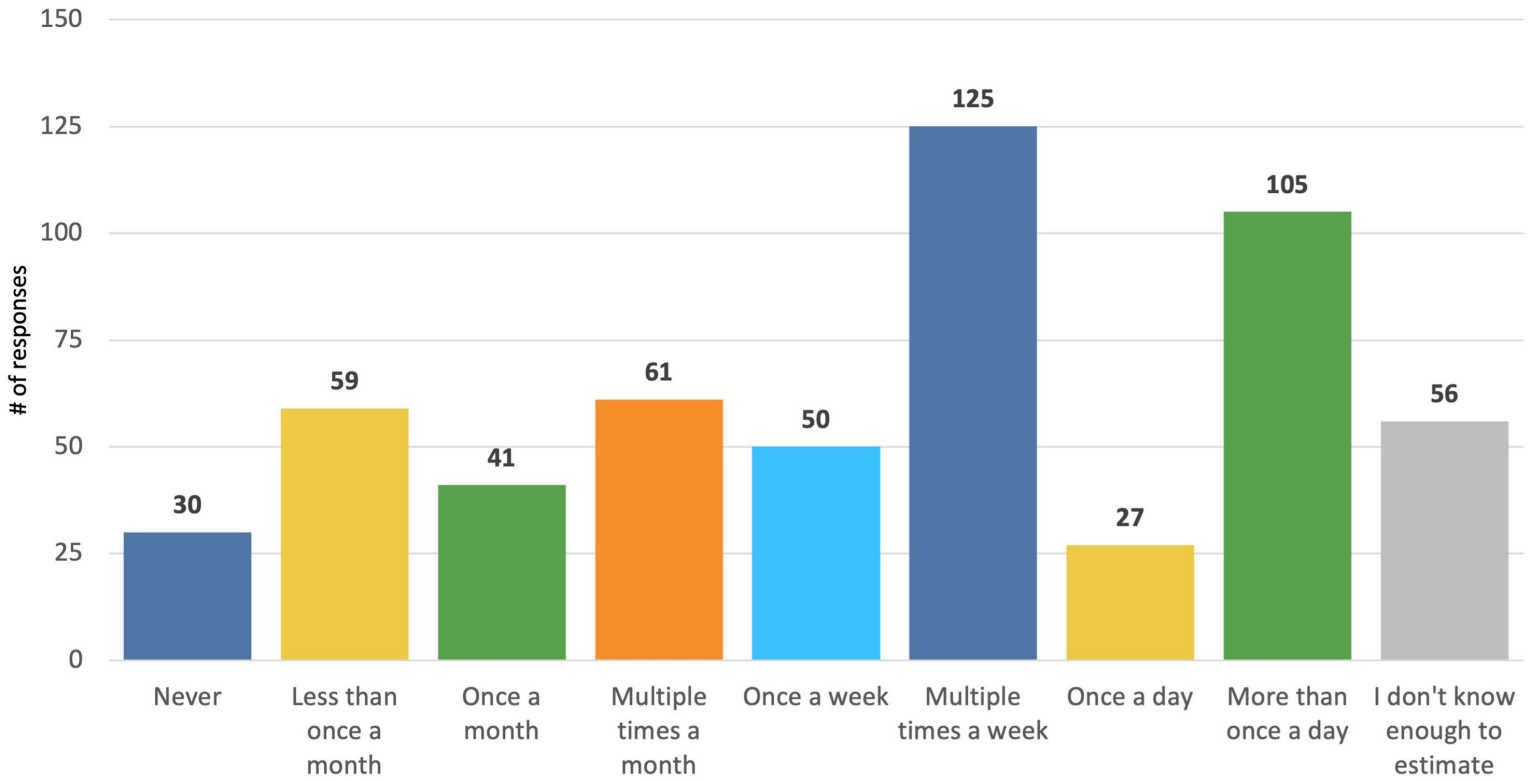
Responses among DVMs and veterinary technicians to the survey question: “In the past year, on average, how often does your practice have to decline veterinary care for patients because the caretaker cannot afford to pay for treatment”? (*n* = 554).

**Figure 4:**
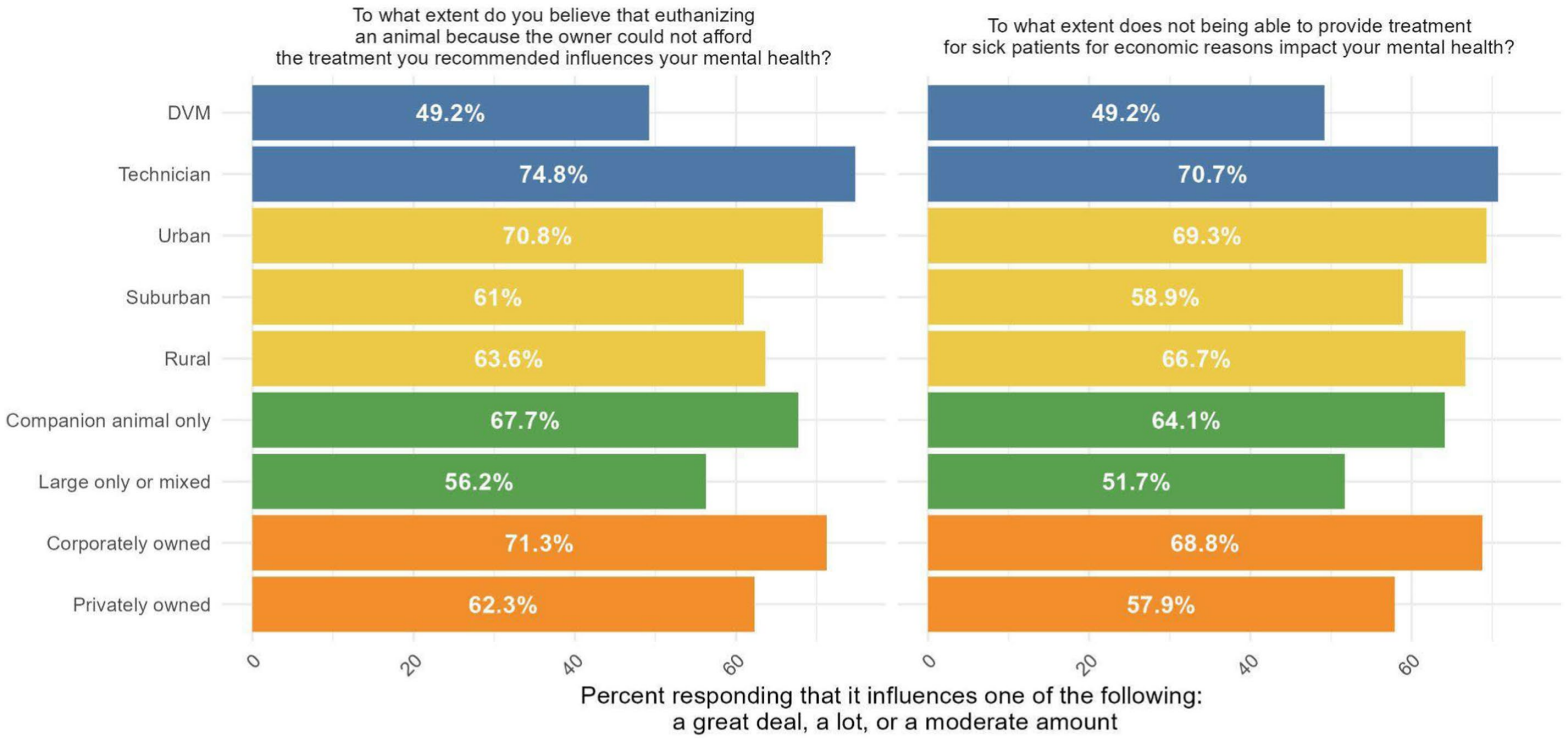
Responses to questions about whether performing euthanasia or having to decline care influences mental health, by type of professional.

Veterinary professionals reported seeing on average between 10–20 clients per day, depending on the type of practice (Figure 2). When asked how often their clinic, on average, has had to divert clients because they could not fit them into their schedule or address their condition in a reasonable time frame, 71.9% (77/107) of practice managers/owners, and 67.3% (379/563) veterinary technicians and veterinarians (DVMs) reported at least weekly (i.e., selected weekly or daily). We also found that 55% (307/554) of DVMs/technicians reported that, on average, owners had to decline veterinary care at least weekly because they could not afford to pay for treatment (Figure 3). Of those who reported clients had to decline veterinary care because they could not afford it, 62.9% (325/517) reported that this type of experience negatively impacts their mental health “moderately,” “a lot,” or “a great deal.” When this question was analyzed by where respondents worked, those from corporately owned practices and technicians reported being impacted the most (Figure 4).

Approximately 72% (392/542) of DVMs and technicians reported that their veterinary team has had to euthanize an animal in the past year because the owner could not afford the recommended treatment and a different decision would have been made if the client had sufficient financial resources. Of these DVMs and technicians, 66.2% (257/388) reported this type of experience negatively impacts their mental health “moderately,” “a lot,” or “a great deal.” When this question was analyzed by where respondents worked, those from corporately owned practices and technicians reported being impacted the most (Figure 4).

### 3.3 Veterinary professional survey—workforce challenges related to veterinary technicians reported

On average, practice managers/owners and DVMs reported having 1.8 CVTs per veterinarian in their clinic. These respondents also reported that approximately 3 (i.e., 2.9) would be the ideal number of CVTs per veterinarian in their clinic to maximize efficiency and number of patients treated. A total of 78.2% (147/188) of DVMs and practice managers/owners “somewhat” or “strongly” agreed with the statement that “CVTs are difficult to find.” Further, when asked how often DVMs perform duties that CVTs could perform, 6/204 (3%) said never, 41/204 (20%) said rarely, 72/204 (35%) said sometimes, and 85/204 (42%) said often.

### 3.4 Pet owner survey—challenges obtaining veterinary care reported

**Figure 5:**
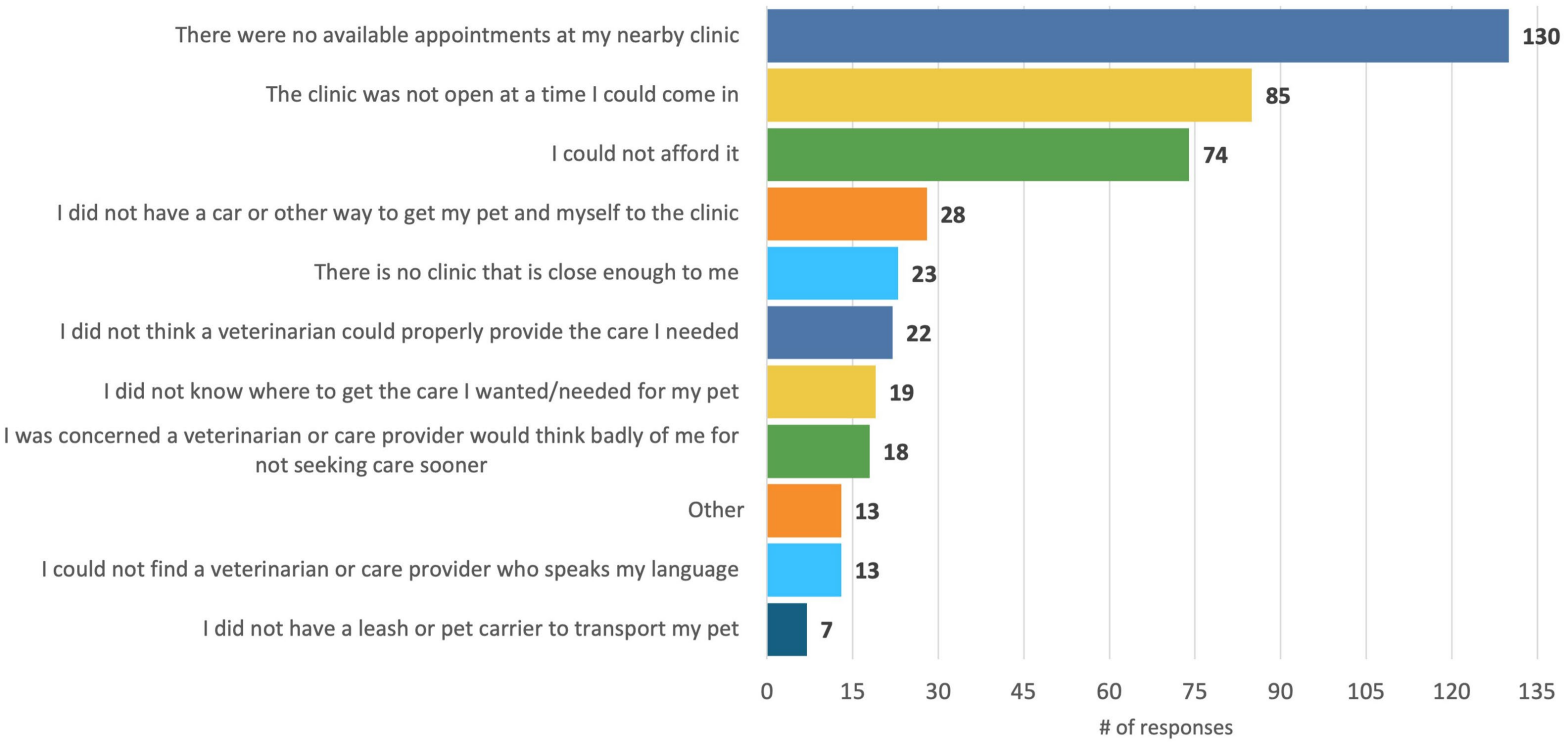
Qualtrics representative pet owner survey responses to the question, “When you tried to see a veterinarian but were unable to, what were the reasons you were unable to get the care you wanted?” This question was asked of the 261/919 respondents who reported that there had been time in the last two years where they tried to see a veterinarian but were unable to.

In the Qualtrics representative survey, 261/919 (28.4%) of pet owners and 51.8% (134/264) of respondents from the in-person survey reported that they had experienced a time in the past two years where they tried unsuccessfully to access veterinary care. Those who reported that they experienced these barriers were asked to indicate the reasons for not being able to obtain care and what type of care they were trying to receive. Qualtrics respondents reported the most common reasons for not being able to obtain veterinary care were “no available appointments at my nearby clinic” (130/261, 49.8%), followed by “the clinic was not open at a time I could come in” (85/261, 32.6%), and “I could not afford it” (74/261, 28.4%) (see Figure 5). Pet owners from the in-person survey reported their most common barriers were “I could not afford it” (164/198, 82.8%) followed by “there were no available appointments at my nearby clinic” (41/198, 20.7%). The most common type of care pet owners from both surveys were seeking was emergency care (online representative survey: 84/261, 32.2%; in person survey: 75/179, 41.9%).

**Figure 6:**
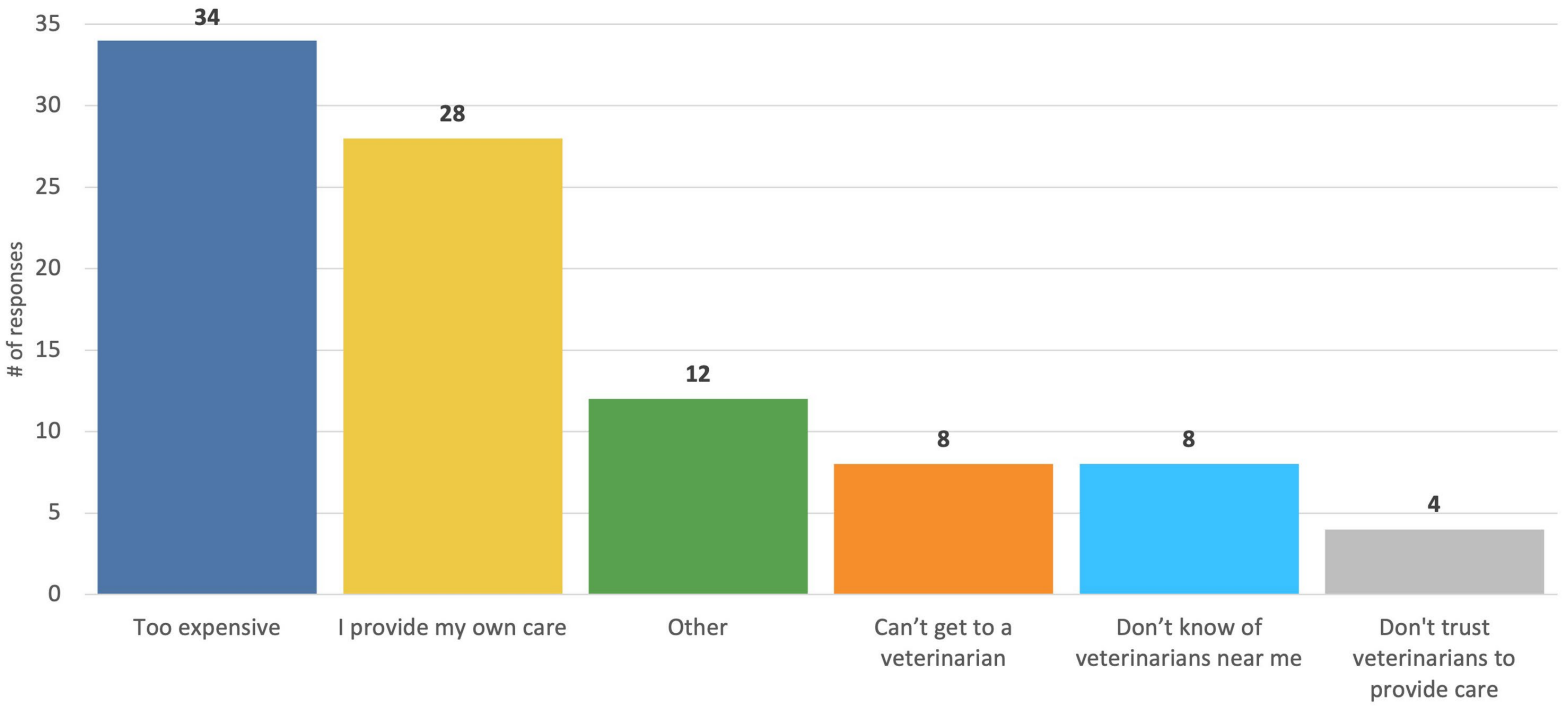
Qualtrics representative pet owner survey responses to the question, “What are some reasons why you have never seen a veterinarian?” This question was asked of the 77/919 respondents who reported that they had ever taken their pet to a veterinarian for any reason.

In the Qualtrics representative survey 77/919 (8.4%) of pet owners and 40/285 (14.0%) of respondents from the in-person survey have never obtained veterinary care. The most common reason why pet owners had never taken their pet to a veterinarian in the representative survey was that it was “too expensive” (See Figure 6; 34/77, 44.2%).

A total of 246/914 (26.9%) of pet owners from the Qualtrics representative survey and 79/285 (27.7%) of respondents from the in-person survey reported having needed to rehome one or more pets in the past to another person or animal shelter. Pet owners from the in-person survey reported the “costs of veterinary care” as the most common answer (31/85, 36.5%) for why they had to give their pet away, while those in the online survey reported “Moved to location that did not allow pets” (84/246, 34.1%).

### 3.5 Veterinary professional perspectives on potential programs and policy solutions

#### 3.5.1 Clarification of roles and creating career opportunities for veterinary technicians

**Figure 7:**
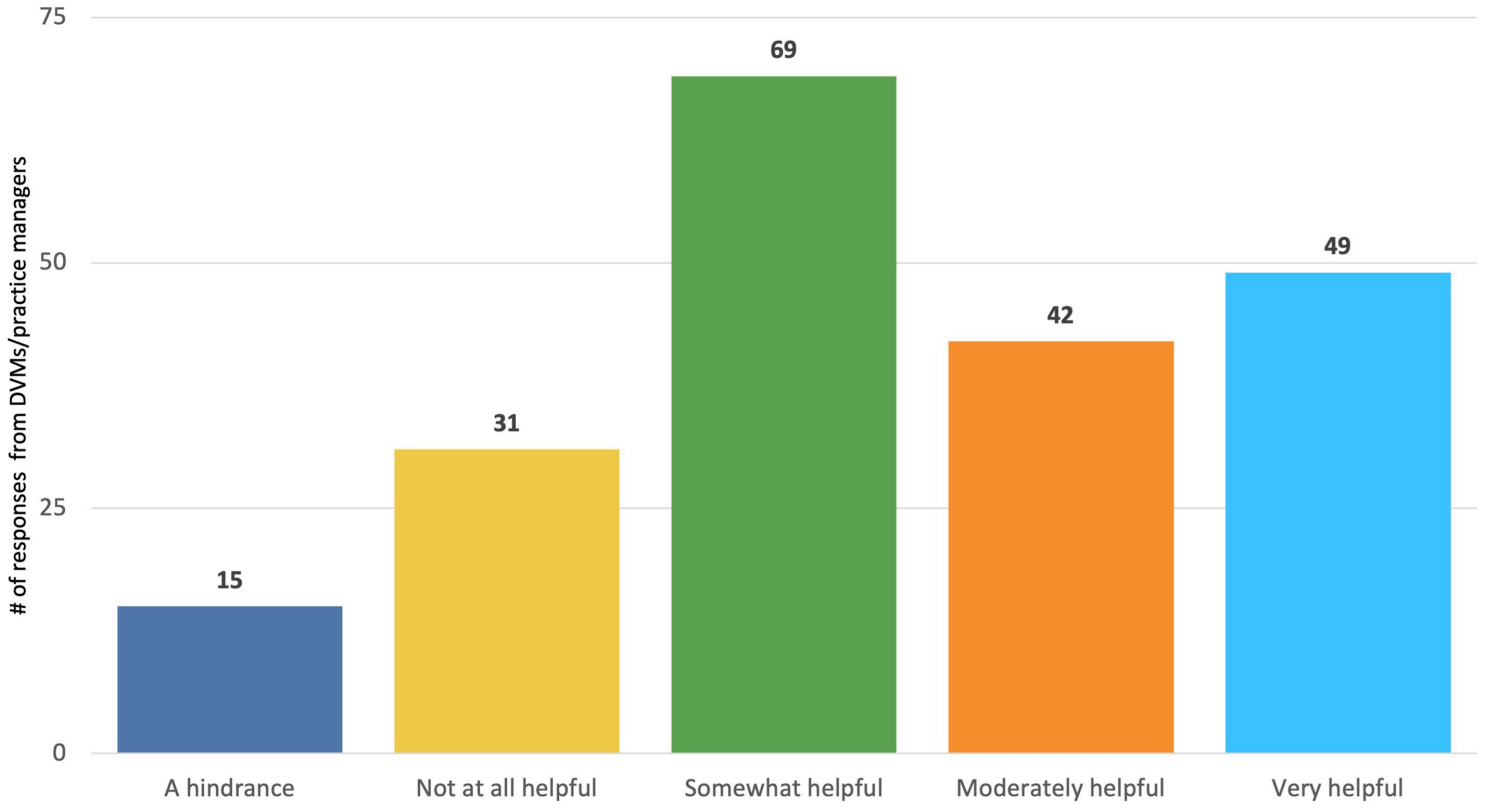
DVM and practice owner/manager responses to the question, “some discussions have occurred about the potential for policy clarifying what tasks are appropriate for delegation under specific levels of supervision by veterinarians to CVTs/RVTs. How helpful would this be for you to more efficiently work with the veterinary technicians in your practice?

**Figure 8:**
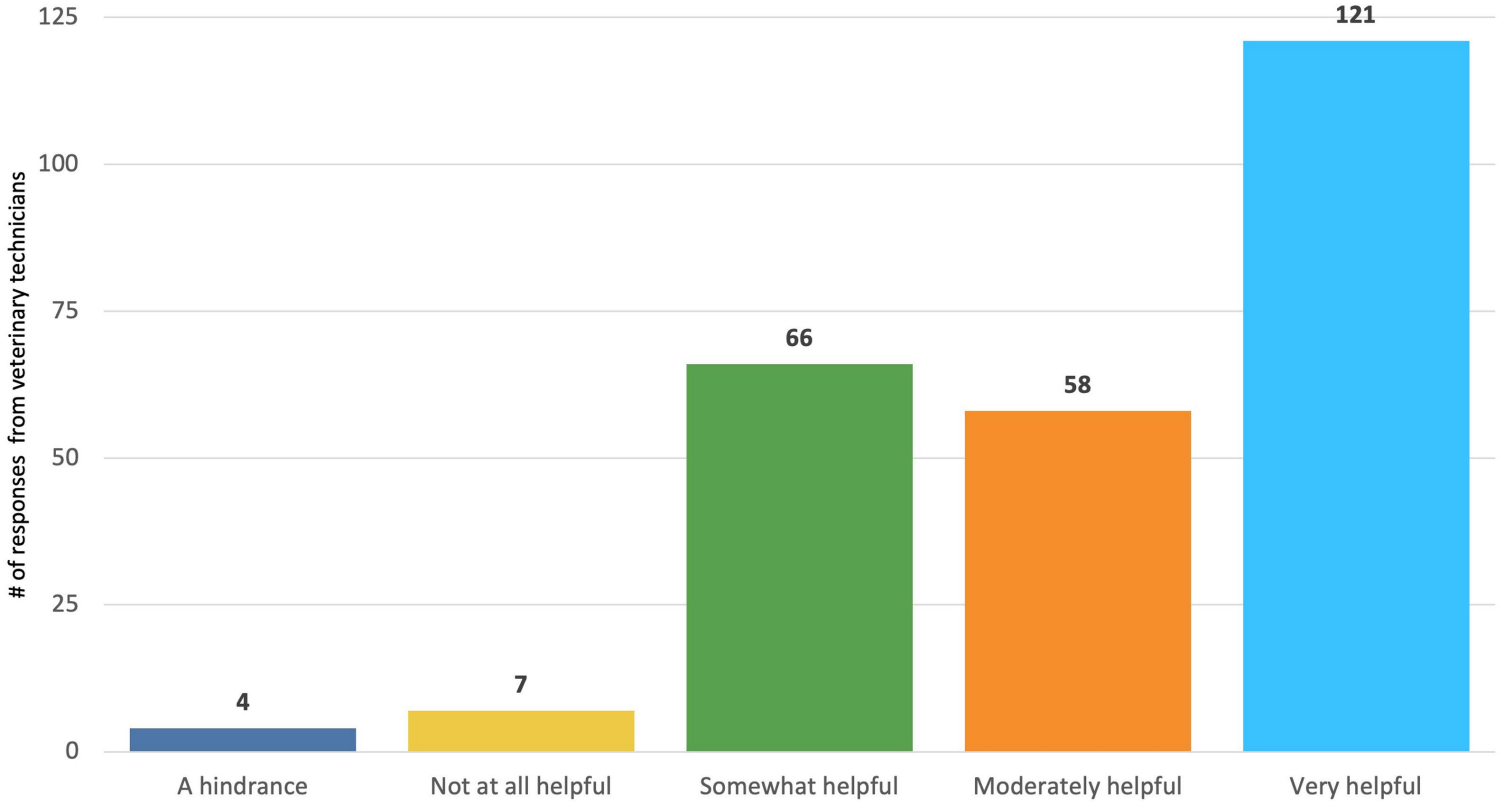
Veterinary technicians’ responses to the question: “Some discussions have occurred about the potential for policy clarifying what tasks are appropriate for delegation under specific levels of supervision by veterinarians to CVTs/RVTs. How helpful would this be for you to more efficiently work with the veterinarians in your practice?”

The majority of DVMs and practice managers/owners (160/206, 77.6%) and technicians (245/256, 95.7%) reported that a policy clarifying what tasks are appropriate for delegation under specific levels of supervision by veterinarians to CVTs would be “somewhat,” “moderately,” or “very” helpful (Figures 7, 8). While there was strong agreement among the majority of respondents that such a policy would be helpful, CVTs had significantly higher perceptions of how helpful it would be than DVMs (CVT mean = 3.575269, DVM mean = 2.893443, *t*(174) = 5.7285, *p* < 0.001).

DVMs and practice owners/managers were asked if they currently employ any Veterinary Technician Specialists (VTS). In response, 26/205 (13%) reported “yes” and 179/205 (87%) reported “no.” When asked if more CVTs obtaining a VTS certification would positively benefit the profession, 97/191 (50.8%) of DVMs/practice managers responded “yes,” 33/191 (17%) responded “no,” and 61/191 (32%) responded “not sure.” When technicians were asked the same question, 167/257 (64.9%) responded “yes,” 33/257 (13%) responded “no,” and 57/257 (22%) responded “not sure.” CVTs had significantly higher perceptions that this would positively benefit the profession than DVMs/practice managers (CVT mean = 2.521, DVM mean = 2.335, *t*(396) = −2.6431, *p* = 0.0085).

**Figure 9:**
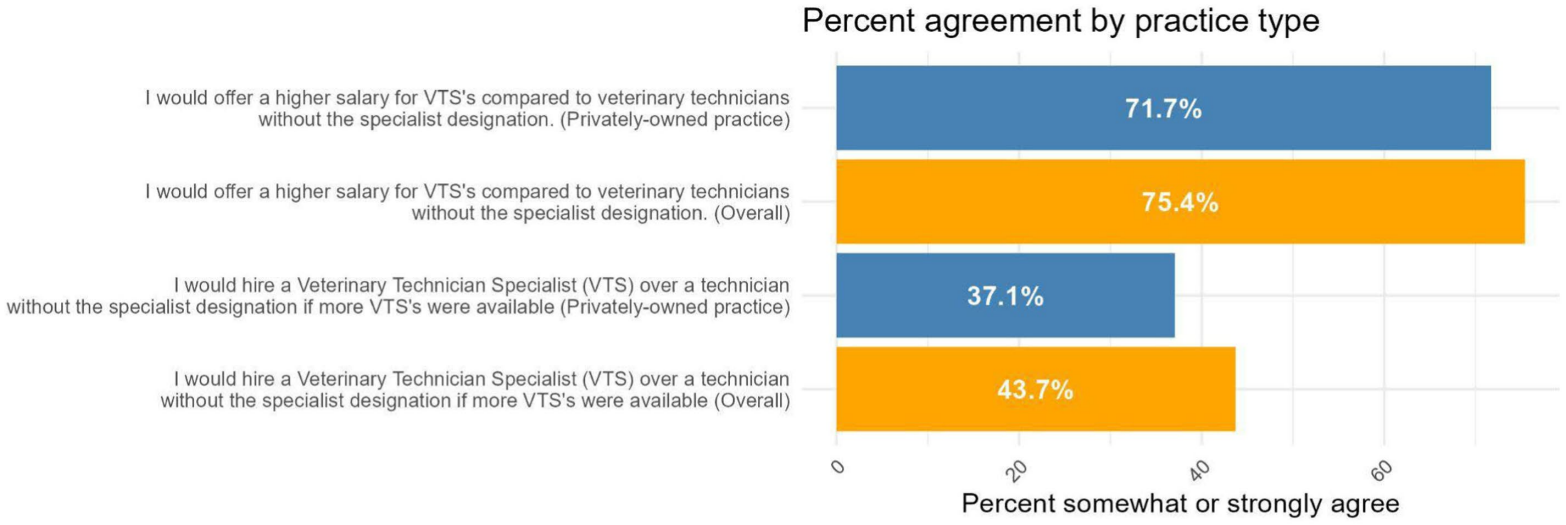
DVM/practice owner/managers’ perceptions of hiring and salary overall and among those in privately owned practices.

**Figure 10:**
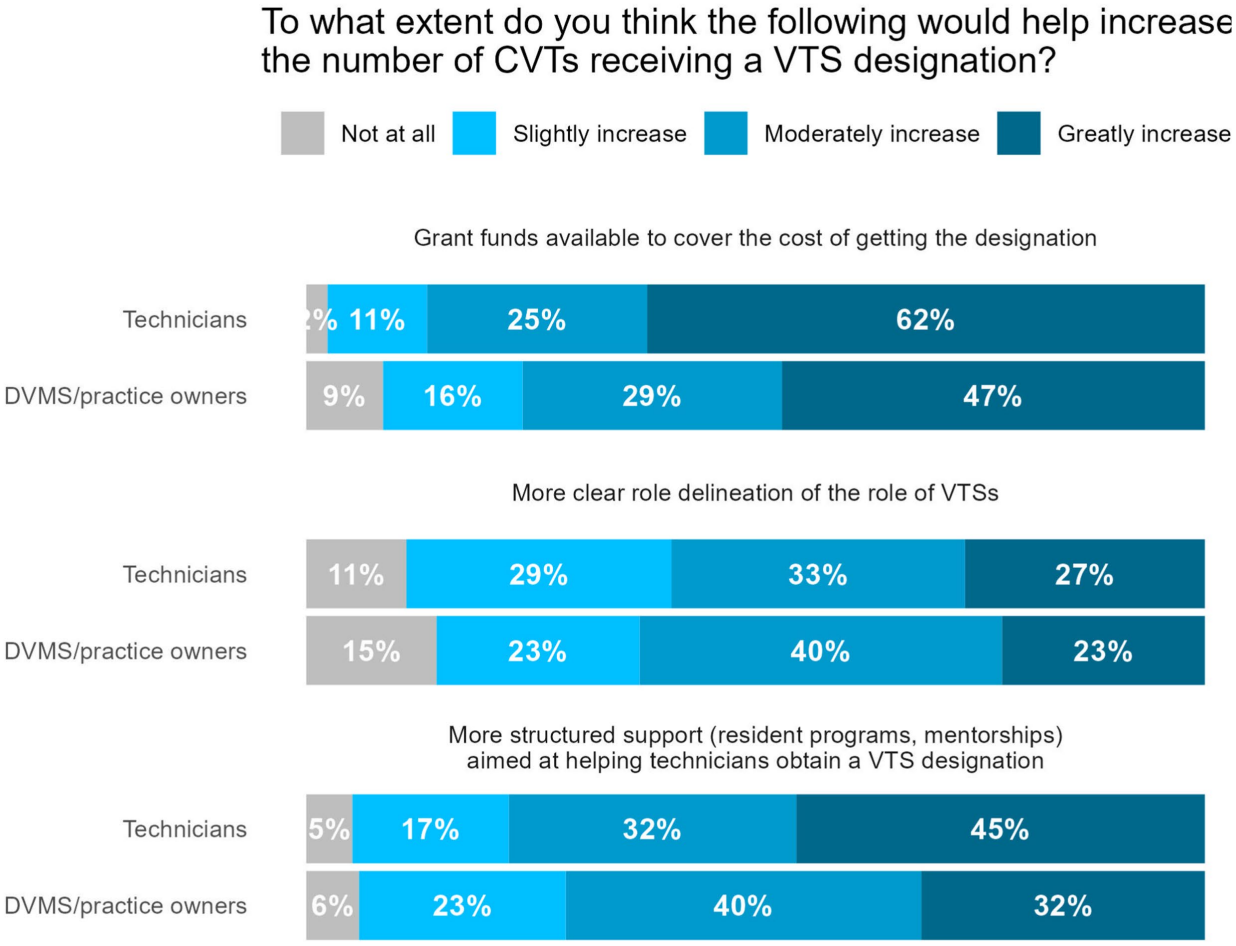
Veterinary professional respondents’ beliefs on the extent to which the following programs would increase the number of Certified Veterinary Technicians (CVTs) receiving a Veterinary Technician Specialist (VTS) designation.

DVMs and practice owners/managers were asked to agree or disagree with the statement: “I would hire a Veterinary Technician Specialist (VTS) over a technician without the specialist designation if more VTS’s were available.” In response, 11/167(7%) responded “strongly disagree,” 22/167 (13%) responded “somewhat disagree,” 61/167 (37%) responded “neither agree nor disagree,” 47/167 (28%) responded “somewhat agree,” and 26/167 (16%) responded “strongly agree.” DVMs and practice owners/managers were asked to agree or disagree with the statement: “I would offer a higher salary for VTS’s compared to veterinary technicians without the specialist designation.” In response, 5/175 (3%) responded “strongly disagree,” 9/175 (5%) responded “somewhat disagree,” 29/175 (17%) responded “neither agree nor disagree,” 69/175 (39%) responded “somewhat agree,” and 63/175 (36%) responded “strongly agree.” Responses were similar among only those who work in privately owned practices and thus may have more autonomy over these decisions (Figure 9). When asked about potential programs/resources to increase the number of CVTs pursuing the VTS designation, grant funds to help cover the cost of the designation were reported as the most helpful (Figure 10).

#### 3.5.2 Grant/voucher programs

Respondents were asked, “Consider a hypothetical grant program for clinics. The program could provide funds to private and non-profit clinics and organizations in your community to increase veterinary service for underserved populations of animals and people. The funds could be used in a variety of ways (e.g., angel funds, vouchers for owners to receive veterinary care, funding for mobile or low cost clinics or bringing veterinary services to the community, investing in telehealth, etc.). To what extent would you be interested in your practice or organization participating in such a grant program?” In response, 69.1% (374/541) of respondents reported that they would be “moderately,” “very” or “extremely” interested in participating. When asked what they would use grant funds for, answers included covering the costs of care for low-income clients, opening up a low-cost ER, providing vaccinations or preventative care clinic in their community, providing free/low cost dental and spay/neuter services for their community, and increasing pay of veterinary technicians.

Respondents were told, “In Rhode Island, a program was founded in 2013 that provides the economic incentive of $125 vouchers to income-qualified pet owners. Income qualified pet owners are approved through the program, rather than through veterinary clinics. The vouchers can be used by pet owners to reduce the costs of receiving veterinary services.” When asked if they or their practice would be willing to accept the vouchers as part of payment for services if a similar program was implemented in their community, 130 (53%) responded “yes,” 27 (11%) responded “no,” and 86 (35%) responded “maybe.” For those who said they would participate in a voucher program, approximately half (66/130) reported that their practice would be able to subsidize a portion of the additional costs of care for voucher holders (e.g., waiving the cost of performing a wellness exam), while 63/130 (48%) reported that they would not.

#### 3.5.3 Loan repayment programs

Respondents were told the following before being asked a series of questions about loan repayment programs: “Knowing that there are federal and state loan forgiveness or repayment programs for veterinary professionals working in designated veterinary shortage areas, we are interested in your perspective on expanding potential loan forgiveness programs to include veterinary technicians and veterinary professionals working in low cost or shelter clinics.” The majority of respondents (351/504, 69.6%) “somewhat” or “strongly” agreed that the development of state-wide student loan repayment assistance programs for veterinary professionals who commit to working in low cost or shelter clinics for a period of time would increase access to veterinary care for underserved populations. Similarly, the majority of respondents (314/502, 62.5%) “somewhat” or “strongly” agreed that the development of statewide student loan repayment assistance programs for CVTs would increase access to veterinary care for underserved populations.

**Figure 11:**
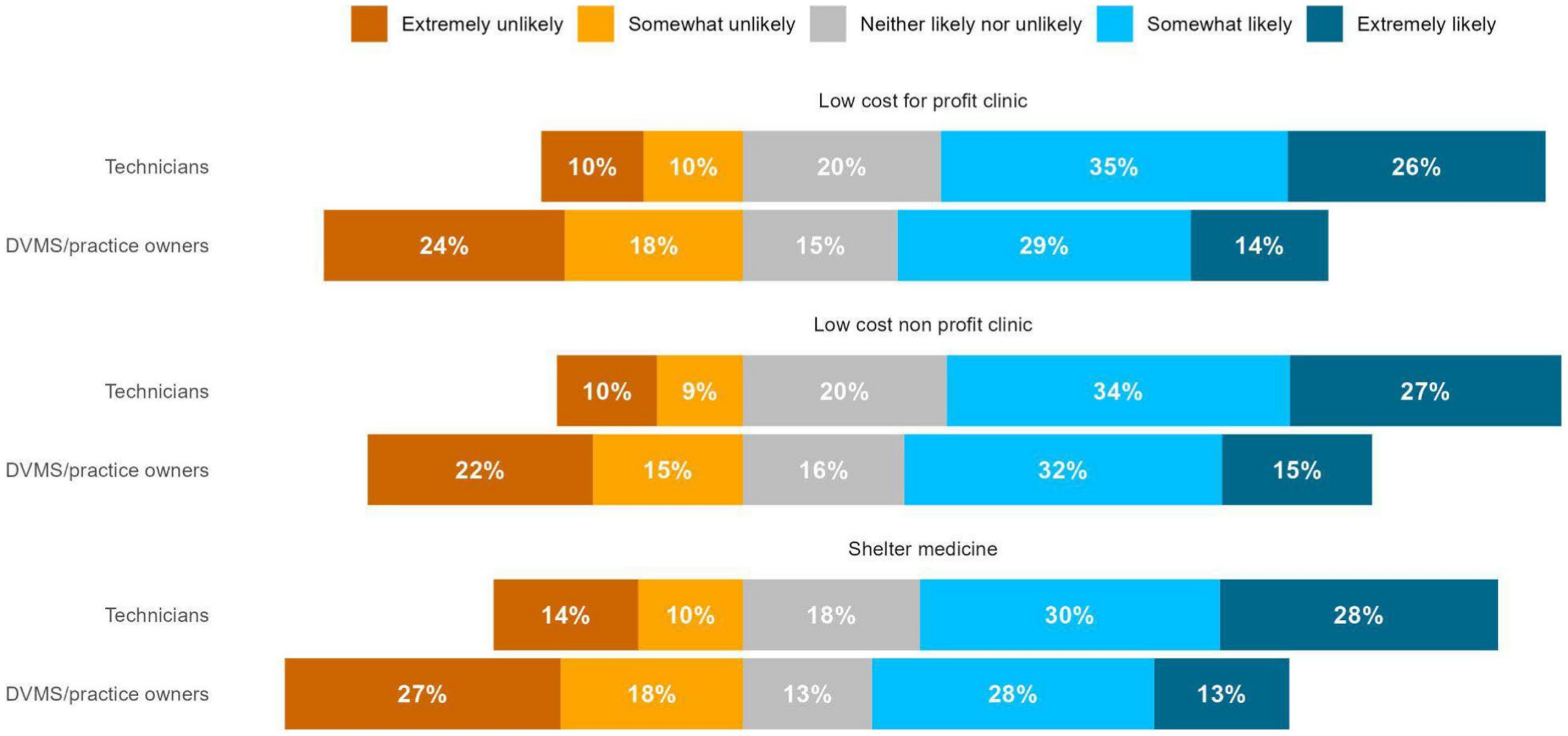
DVM and veterinary technician’s responses to the question: “how likely would you be (or would you have been at the beginning of your career) to work in the following settings to provide care to underserved pets if there was also a state-wide loan repayment assistance program for veterinary professionals who commit to working there for at least 3 years?”

Respondents were asked if they would have been likely to work in a low-cost for-profit clinic, low-cost non-profit clinic, or shelter medicine if there was a state-wide loan repayment assistance program for veterinary professionals who commit to working in these types of organizations for at least 3 years. In response, the majority of technicians reported they would be “somewhat” or “extremely” likely to work for a low-cost non-profit clinic (61.2%), a low-cost for-profit clinic (60.2%), and shelter medicine (57.6%) (Figure 11), while fewer than 50% of DVMs reported they would (46.6, 42.9, and 41.5% respectively) (Figure 11).

#### 3.5.4 Veterinary professional associate

The questions pertaining to VPAs were preceded by the following description: “In the next few questions, we would like to know your perspective on the potential introduction of a mid-level practitioner, or veterinary professional associate (VPA) into the profession. There has been an effort to develop a Masters of Veterinary Clinical Care (MSB-VCC) degree program to train such veterinary professional associates. Graduates of a program like this would be trained in clinical case management and would work under the supervision of a veterinarian, who determines the level of appropriate delegation.”

**Figure 12:**
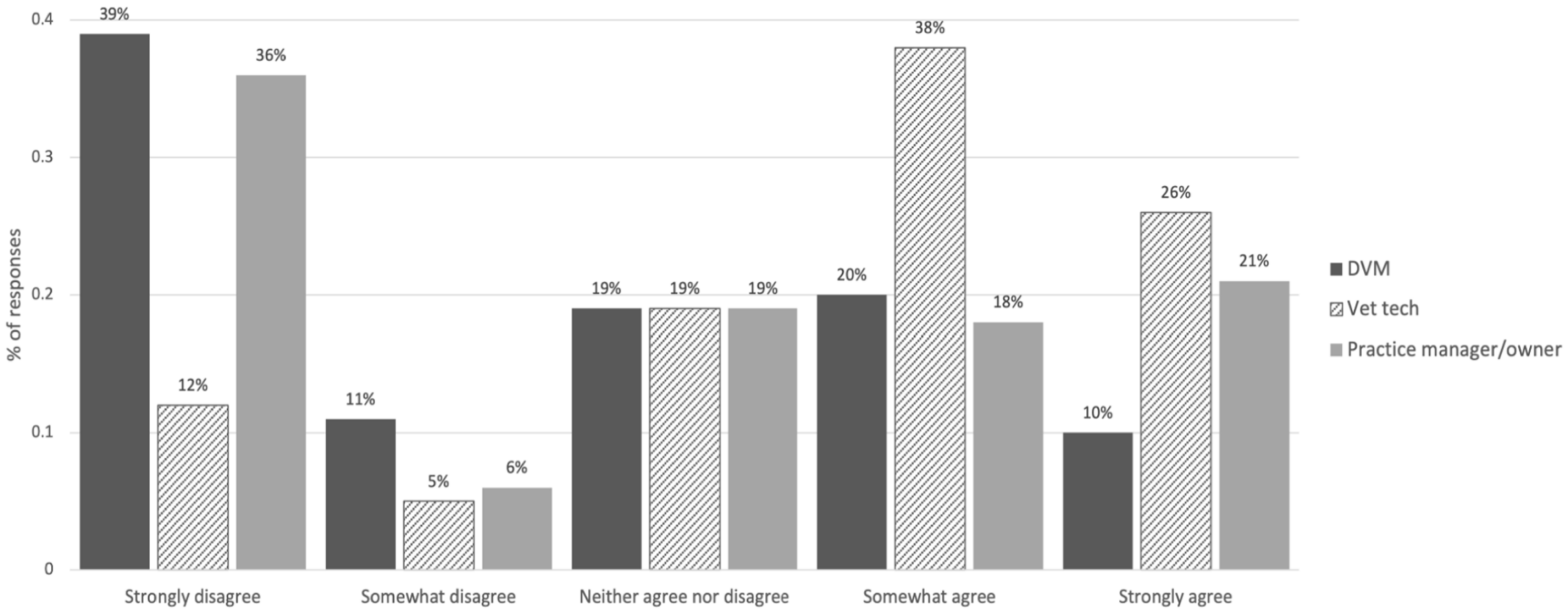
Veterinary professionals’ agreement with the statement “The development of a ‘mid-level’ veterinary professional associate (VPA) through a Masters of Veterinary Clinical Care degree would increase access to veterinary care for underserved populations”.

**Figure 13:**
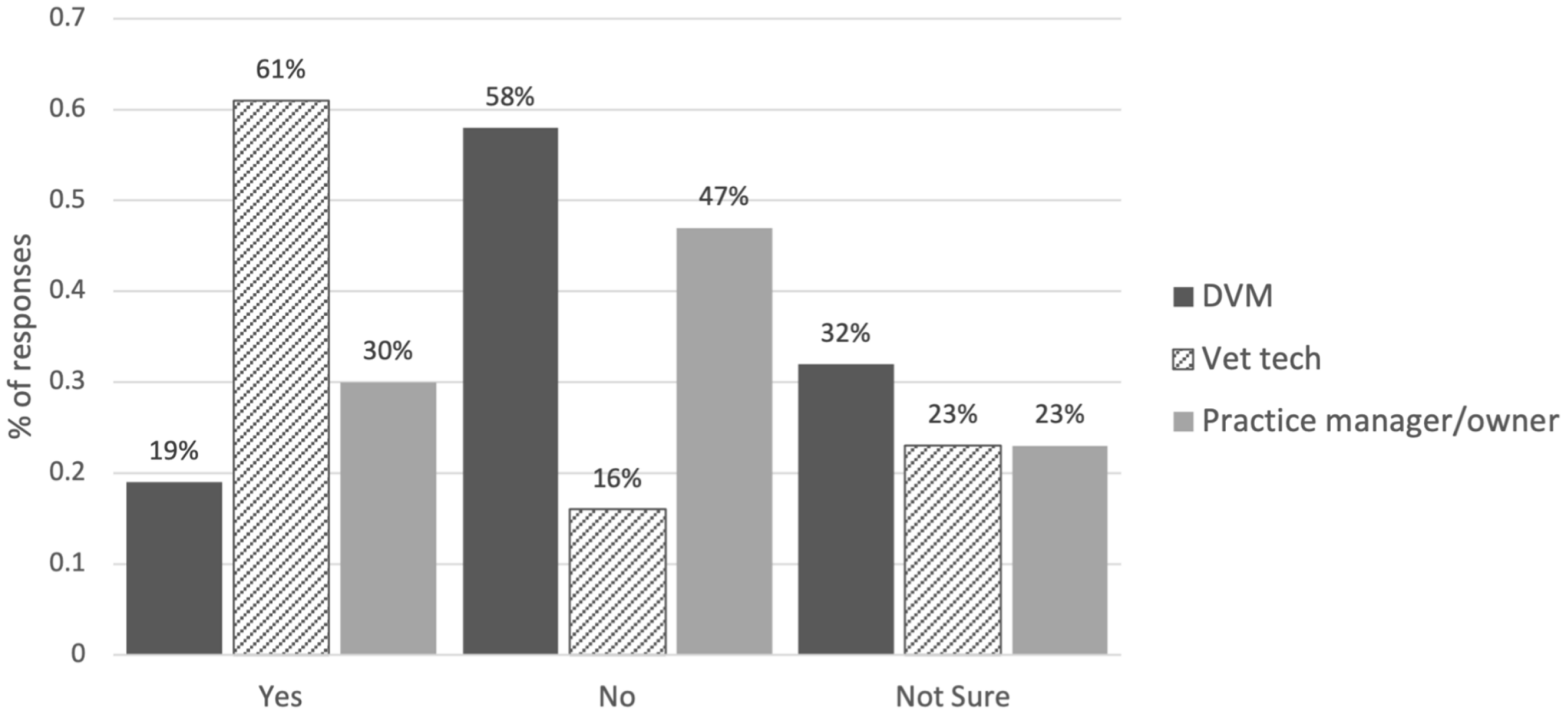
Veterinary professionals’ responses to the question: “Overall, do you think a VPA would positively benefit the profession?”

A total of 45.9% (195/424) of respondents reported that a VPA would positively benefit the profession, 41.1% (174/423) of respondents reported that a VPA would positively benefit their practice, and 51.2% (218/425) of all respondents “somewhat” or “strongly” agreed that the development of a VPA position would increase access to veterinary care for underserved populations (Figures 12, 13). Beliefs about the VPA differed between different types of professionals; the majority of veterinary technicians believed that the development of the VPA position would positively impact the profession (61%) and increase access to veterinary care (64%), but less than 50% of DVMs and practice owners/managers believed this (Figures 12, 13).

When asked why respondents supported or opposed the development of a VPA position, selected reasons given for supporting a VPA position included:

- Reduction of DVM stress
- Allow practices to see more patients and be more efficient
- Improve the mental health and work-life balance of veterinary professionals
- Save clients money by providing some services at a lower cost
- Provide new career pathway for CVTs interested in more schooling and expanding their roles
- Increase recruitment of those interested in the veterinary field through a position that requires less debt than veterinary school
- Allow DVMs to focus on critical cases and surgeries in emergency settings
- Help address veterinary shortages, especially in rural areas

Selected reasons given for opposing development of a VPA included:

- Already have CVTs that currently or could fill this role with more training and better utilization
- Increase pay and retention of CVTs before creating a new role
- Fear that corporate practices will use this to increase profits by trading veterinarians for multiple, lower paid VPAs
- May further increase DVM shortage
- No indication that VPAs would work in underserved areas, especially when large corporations will offer better pay
- Concerns about DVM liability related to having a VPA practice under their license

#### 3.5.5 Telemedicine

**Figure 14:**
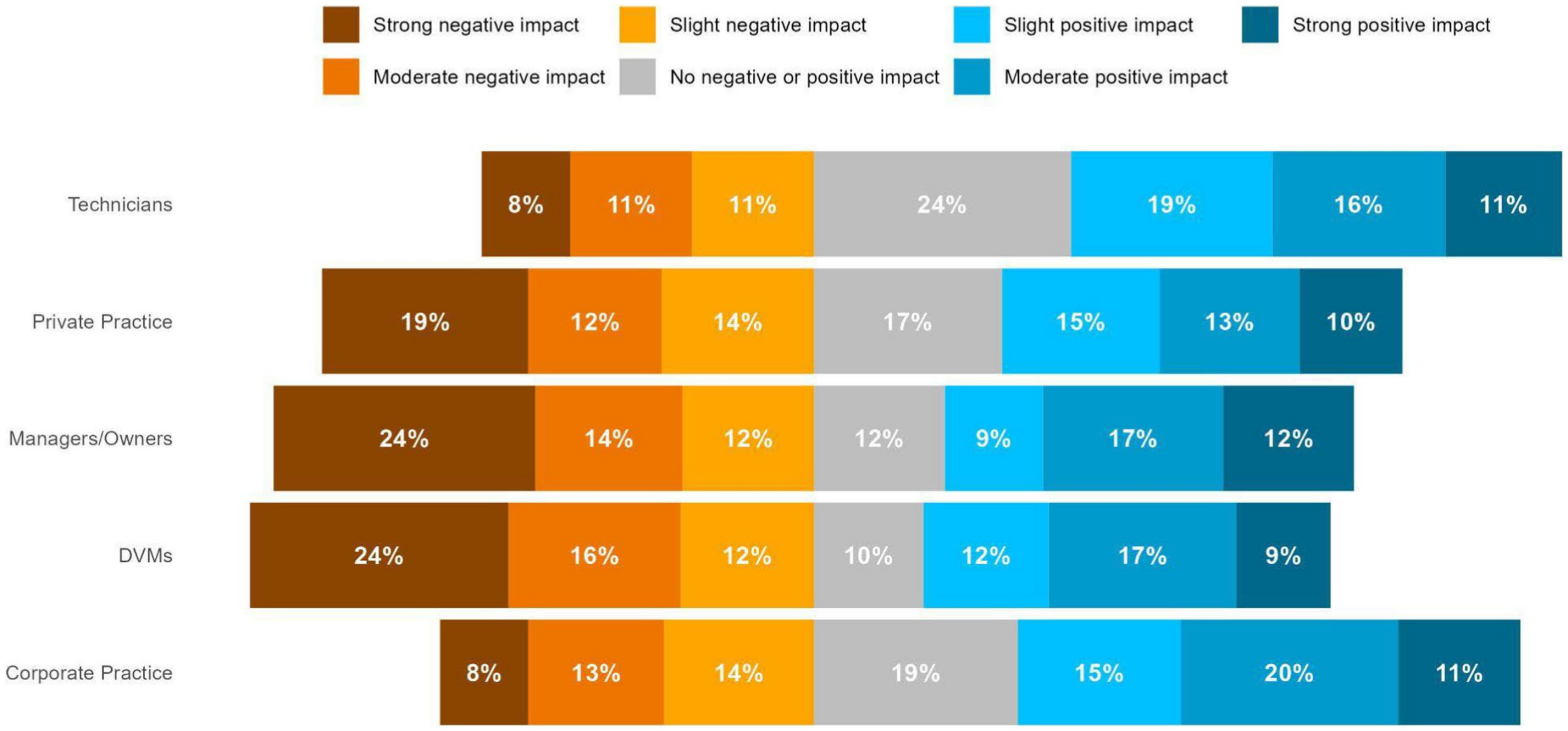
Veterinary professionals’ responses to the question: “In May 2023 Senate Bill 1,053 was signed into law in Arizona which allows veterinarians licensed in Arizona to establish a veterinarian-client-patient relationship (VCPR) through telemedicine. Veterinary professionals and stakeholders may have different perspectives on this idea of establishing a VCPR through telemedicine, so we’d like to better understand your perspective on this topic. To what extent do you think a similar law in Colorado would positively or negatively impact the profession?”

The majority (269/424, 63.4%) of respondents “somewhat” or “strongly” believed that expanding the use of telemedicine would increase access to veterinary care for underserved populations. More than half of respondents (213/415, 51.3%) never used telemedicine or used telemedicine less than once a month, and the most common way telemedicine was currently used was for follow-up. Approximately 43% (172/404) of respondents reported that they believed a law allowing veterinarians to establish a veterinarian-client patient relationship (VCPR) virtually, or through telemedicine would have a “slight,” “moderate,” or “strong” positive impact on the profession, while 39% (158/404) reported that they believed it would have a “slight,” “moderate,” or “strong” negative impact on the profession (74/404, 18% reported no impact). Responses to this question varied by type of profession and practice, with positive beliefs slightly more prevalent among technicians and those working in corporate practices (Figure 14). Over half (52%, 211/405) of respondents “somewhat” or “strongly” agreed that the ability to establish a virtual veterinarian-client patient relationship (VCPR) through telemedicine would increase the amount of care that veterinary professionals could provide to underserved populations.

### 3.6 Pet owner perspectives—potential programs and policy solutions

All 18 programs or solutions offered as ways to potentially increase access to care were ranked by the majority of pet owners as helpful (Table 2). However, the solution ranked as being the most helpful by respondents in both pet owner samples was a new low-cost clinic that can provide sick and emergency care (Qualtrics online: 74.6%, In-Person: 91.4% rated as VEH, or “Very” or “Extremely” Helpful). The second most highly rated program in the online sample was a program (e.g., Aligncare) for income-qualified pet owners that pays 80% of the costs of veterinary visits, and pet owners pay 20% (Qualtrics online: 71.4%, In-Person: 88.6% VEH). The third most highly rated program in the in-person sample was a program that provides income-qualified pet owners with vouchers to receive a discount on veterinary services at a nearby clinic (Qualtrics online: 68.6%, in-person: 90.7% VEH). Other highly rated solutions included more availability of veterinary appointments outside of traditional working hours (Qualtrics online: 69.8%, In Person: 81.8% VEH); funds that can be applied to cover a portion of the costs of a veterinary visit (Qualtrics online: 68.7%, In Person: 90.2% VEH); a new low-cost clinic in your community that can provide preventative care services for pet owners (Qualtrics online: 69.8%, In Person: 89.4% VEH); and affordable pet health insurance options (Qualtrics online: 69.7%, In Person: 79.6% VEH). The least helpful solutions according to ratings included information on where to access veterinarians who speak languages other than English (Qualtrics online: 14.3%, In Person: 20.8% rated as “Not at all Helpful”), allowing pet owners to bring pets on public transportation for a scheduled or emergency veterinary appointment (Qualtrics online: 9.9%, In Person: 15.6% rated as “Not at all Helpful”), and ride-shares (e.g., uber, lyft) allowing pets in cars with their owners when going to a veterinary visit (Qualtrics online: 9.4%, In Person: 14.1% rated as “Not at all Helpful”).

**Table 2:** Responses of pet owners from the representative survey to the question of: “Rank the extent to which the following services would be helpful to you or others in their community facing problems getting the care they want or need for their pet”.

Proposed program/policy	Number and % selected “very” or “extremely” helpful	Number and % selected “not at all” helpful
More mobile (traveling)/pop-up veterinary clinics providing preventative care services (e.g., vaccines or shots, dental care) and spay/neuter services in your community on certain days of the month	469/917 (51.1%)	38/917 (4.1%)
A new low-cost clinic in your community that can provide preventative care services for pet owners	639/916 (69.8%)	27/916 (2.9%)
A new low-cost clinic in your community that can provide sick and emergency care for pet owners	684/917 (74.6%)	22/917 (2.4%)
Allowing pet owners to bring pets on public transportation for a scheduled or emergency veterinary appointment	488/916 (53.3%)	91/916 (9.9%)
Ride-shares (e.g., uber, lyft) allowing pets in cars with their owners when going to a veterinary appointment	478/916 (52.2%)	86/916 (9.4%)
A program that provides income-qualified pet owners with vouchers to receive a discount on veterinary services at a nearby clinic.	628/916 (68.6%)	38/916 (4.1%)
A program for income-qualified pet owners that pays 80% of the costs of veterinary visits, and pet owners pay only 20%	653/915 (71.4%)	38/915 (4.2%)
Easy access to information on and help with signing up for pet insurance	505/915 (55.2%)	53/915 (5.8%)
Funds that you can apply for to cover a portion of the costs of your veterinary visit	631/918 (68.7%)	33/918 (3.6%)
Affordable pet health insurance options	636/913 (69.7%)	33/913 (3.6%)
Vaccine clinics at your local animal shelter	632/917 (68.9%)	33/917 (3.6%)
Information on where to access veterinarians that speak languages other than English	384/916 (41.9%)	131/916 (14.3%)
More availability of pet food pantries in your community	525/917 (57.3%)	53/917 (5.8%)
Vouchers for income-qualifying pet owners to purchase quality pet food at a discounted price	581/915 (63.5%)	47/915 (5.1%)
Guidance on where to shop for low cost pet foods and free pet food delivery	541/918 (58.9%)	54/918 (5.9%)
Availability of telemedicine options, where you can meet with a veterinarian on a phone/video rather than going to a clinic in person	514/917 (56.1%)	55/917 (6.0%)
More availability of veterinary appointments in general	562/916 (61.4%)	33/916 (3.6%)
More availability of veterinary appointments outside of traditional working hours (8–5, Monday through Friday)	640/917 (69.8%)	29/917 (3.2%)

We also asked pet owners in the online survey about their perspective on telemedicine and seeing veterinary professionals other than DVMs for various procedures. Of the pet owners, 666/919 (72.5%) reported that “Yes” they felt comfortable seeing a veterinarian through a telemedicine appointment. Additionally, 493/665 (74.1%) of those who felt comfortable with telemedicine reported that “Yes,” they would also feel comfortable seeing a veterinarian for the first time via a virtual telemedicine visit. A total of 588/919 (64.0%) of respondents reported that access to telemedicine would “somewhat” or “greatly” increase their likelihood of contacting a veterinarian.

We asked pet owners about their comfort seeing a veterinary professional other than a DVM, including a CVT, VTS, or VPA. Pet owners were provided with an explanation of each type of professional and the education/training provided to each type of professional and then asked whether they would feel comfortable seeing each professional for a variety of different procedures or only a DVM. For example, CVTs were described as similar to a registered nurse in human healthcare; VTSs were described as veterinary technicians who have additional training in a specified field of veterinary medicine and pass an additional competency exam; and VPAs were described as a new type of veterinary professional that some have proposed creating that would be similar to a physician’s assistant (PA) in human healthcare and would graduate from a Masters of Veterinary Clinical Care degree program (See Supplemental materials for the full wording). In response, the majority of respondents indicated they felt comfortable having a CVT perform vaccine administration (58.1%) and treatment of non-urgent sickness/medical conditions (52.1%; Table 3). Respondents were less comfortable having a CVT perform spays (21.2%) or neuters (20.2%; Table 3). Pet owners’ comfort level with having VPAs perform procedures generally mirrored their comfort level with a CVT; however, fewer reported feeling comfortable with VPAs performing less invasive/urgent procedures (e.g., annual check-ups, vaccine administration, treatment of less urgent conditions) while more respondents reported feeling comfortable with VPAs treating urgent conditions and more invasive procedures (e.g., dental surgery, spaying, neutering, and treatment of urgent conditions) when compared to a CVT (Table 3).

**Table 3:** Qualtrics selected pet owners’ responses to whether they would feel comfortable having various professionals provide for their pet instead of a veterinarian.

Service	# and % selecting registered veterinary technician (RVT)	# and % selecting veterinary technician specialists (VTS)	# and % selecting veterinary professional associate (VPA)	# and % selecting only veterinarian (DVM)	# and % selecting “not sure”
Annual exam/check-up	446/918 (48.6%)	361/918 (39.3%)	379/918 (41.3%)	288/918 (31.4%)	110/918 (12.0%)
Vaccine administration	529/911 (58.1%)	467/911 (51.3%)	425/911 (46.7%)	204/911 (22.4%)	88/911 (9.7%)
Spaying	193/911 (21.2%)	169/911 (18.6%)	214/911 (23.5%)	533/911 (58.5%)	78/911 (8.6%)
Neutering	184/911 (20.2%)	168/911 (18.4%)	205/911 (22.5%)	540/911 (59.3%)	81/911 (8.9%)
Dental care (e.g., cleaning dirty teeth)	420/911 (46.1%)	453/911 (49.7%)	424/911 (46.5%)	231/911 (25.4%)	89/911 (9.8%)
Dental surgery (e.g., removing teeth)	188/905 (20.8%)	200/905 (22.1%)	219/905 (24.2%)	500/905 (55.2%)	84/905 (9.3%)
Treatment of non-urgent sickness/medical conditions (e.g., skin rashes, ear infections, lameness)	474/909 (52.1%)	451/909 (49.6%)	452/909 (49.7%)	244/909 (26.8%)	75/909 (8.3%)
Treatment of urgent sickness/medical conditions (vomiting, not eating)	279/889 (31.4%)	247/889 (27.8%)	340/889 (38.2%)	444/889 (49.9%)	79/889 (8.9%)

Respondents were given information about the different types of professionals (including the proposed VPA) and were asked to check if they would feel comfortable only having a veterinarian provide the service or if they would feel comfortable having a Registered Veterinary Technician, a Veterinary Technology Specialist, or a Veterinary Professional Associates perform each. Below are the number and percentage of respondents who checked each (out of the total number completing the question).

## 4 Discussion

We surveyed veterinary professionals and pet owners to examine the scope of access to care and workforce challenges in Colorado as well as their perspectives on potential policy solutions and programs to address these challenges. We found that the scope of access to care challenges was similar in Colorado as previous nation-wide surveys: 28% of pet owners in our online, representative sample had experienced a time in the past two years where they tried to access care but could not, which was the same percentage found in the AVCC’s (1) nationwide survey. While the AVCC survey found that the most common barrier was cost, we found that among our representative sample, a lack of available appointments was the most common barrier followed by the clinic not being open when they could come in and then cost. However, the cost of care was the primary reason why our sample of pet owners recruited in person at pet food pantries and low-cost clinics were unable to access care and why 8% of pet owners in our online sample had never received veterinary care. These findings highlight the importance of continuing to provide a spectrum of care options to meet the financial limitations of clients (24) and provide programs to pet owners to help cover the costs of care. Indeed, we found that programs like Aligncare (2), vouchers, and other funds to help cover the costs of care were ranked as very helpful by the vast majority of pet owners, and the majority of veterinary professionals were interested in participating in grant programs and vouchers to help expand access to care.

We found that veterinary professionals in Colorado frequently have to decline care for patients or euthanize pets due to cost and frequently have to divert clients because they lack sufficient staff. We also found that the majority of professionals reported that not being able to provide treatment or having to euthanize pets due to cost negatively impacted their mental health. These findings support prior work on the personal and professional impacts of workforce challenges on veterinary professionals. In a survey of over 1,000 veterinarians in the United States and Canada, for example, Kipperman et al. (19) found that the majority of respondents reported that their clients’ economic limitations affected their ability to provide the desired care for their patients on a daily basis. Veterinarians have reported in prior research that client economic limitations as well as having too high of a caseload are important contributing factors to their workplace burnout (18–20). Additionally, Holowaychuk et al. (29) found that veterinary emergency care providers had higher rates of burnout, including greater total emotional exhaustion and lower total personal accomplishment scores compared to emergency department human healthcare professionals. The authors argue that one reason why might be the psychological demands associated with having to perform euthanasia and the resulting psychological distress and ethical conflict (30, 31). Our findings provide further evidence to this body of work, which suggests that addressing workforce challenges is a critical component of not just increasing access to veterinary care, but also addressing the significant mental health challenges in the profession. Through keeping the workforce mentally healthy, they might stay in the profession longer, and this alone increases access to care.

We identified several additional policy solutions that were strongly supported by veterinary professionals and the public. We found that DVMs and practice managers generally reported wanting more CVTs than what they currently had and having to often perform the duties of CVTS, and the vast majority reported having difficulty finding more CVTs. Our findings are similar to prior studies on perceptions of veterinary technicians; for example, Shock et al. (32) found that among managing veterinarians and office/business managers in Ontario, Canada, the majority of respondents (80%) found it difficult to hire registered veterinary technicians, and about 1/4 of respondents reported that veterinarians in their practice performed RVT duties often or always. Relatedly, there was strong support reported in our sample of veterinary professionals for policy solutions that involved enhanced recruitment and retention of CVTs. This included policies clarifying what tasks are appropriate for delegation under specific levels of supervision by veterinarians to CVTs [which could potentially increase efficiency in the clinic, enabling for more patients to be seen; see (33, 34)], as well as loan repayment programs for CVTs and programs that could facilitate CVTs obtaining a VTS certification. More than 50% of pet owners also reported that they would feel comfortable seeing a CVT for tasks such as vaccine administration and treatment of non-urgent medical conditions. These findings support the ongoing efforts of some policy makers to better define the roles of technicians [e.g., (23)]; for example, a bill clarifying the roles of technicians was recently introduced into the Colorado legislature (35).

These findings also suggest that policymakers should consider expanding loan repayment programs to include technicians; loan repayment programs for technicians could be especially impactful given the findings of the National Association of Veterinary Technicians in America (NAVTA)’s 2022 survey, which found that the average salary of technicians is $52,000, more than 33% of technicians have student loan debt, and the average student loan debt is $29,700, which is slightly higher than the overall average US student loan of $28,950 (36). Loan repayment programs may be particularly useful for increasing access to care if they focus on providing payments for technicians who work in low-cost or non-profit clinics or shelter situations for several years, as the majority of our sample of technicians reported that they would be somewhat or extremely likely to work in these settings if such programs existed.

Our findings also highlight the value of building opportunities for more CVT to pursue a VTS designation as a way to create further career opportunities. In our sample, few DVMs and practice owners/managers employed VTSs, but the majority of DVMs and practice owners/managers agreed that they would offer a higher salary for VTSs. Further, many (44%) agreed that they would hire a VTS if more were available. The majority of veterinary professionals surveyed believed that more CVTs obtaining a VTS would benefit the profession. Our findings also provide insight into how programs and policies could support the VTS pathway—the majority of our sample believed that more clear role delineation of the role of VTSs, more structured support (resident programs, mentorships) aimed at helping technicians obtain a VTS certification, and grant funds to cover the cost of getting certified would be beneficial in increasing the number of CVTs pursuing the designation. Grant funds were perceived as the most helpful, as 87% of technicians and 76% of DVMs and practice owners/managers believed they would “moderately” or “greatly” increase the number of VTSs. No states to our knowledge have implemented such grant programs, which highlights another policy opportunity for addressing workforce and access to care challenges.

Expanding telemedicine was another policy solution where support was relatively high; the majority of professionals believed that expanding the use of telemedicine would increase access to veterinary care for underserved populations. Additionally, slightly more veterinary professionals believed that being able to establish a virtual VCPR would have a positive impact on the profession than believed it would have a negative impact. The majority of pet owners reported that they would feel comfortable using telemedicine and seeing a veterinarian for the first time via virtual appointment, and felt that telemedicine options would increase their likelihood of seeing a veterinarian. These findings support the results of Smith et al. (37), who found that the majority of pet owners surveyed who had never used telemedicine before indicated that they would be interested in using telemedicine in the future and that vulnerable pet owners were statistically more likely to have used telemedicine. Further, Smith et al. (37) found that one of the top three reasons that veterinary professionals used telemedicine during the COVID-19 pandemic was to increase access to care for high-risk clients.

Increased access to telemedicine could address the second most common reason pet owners in our online representative sample could not get access to care-the clinic not being open at a time they could come in, as well as the barriers of not having a clinic close enough or access to transportation that were also commonly reported (Figure 3). Overall, our findings on telemedicineare relevant given recent policy discussions and decisions on the virtual VCPR; while several states, such as Arizona, Florida, and California, have passed laws enabling a VCPR to be established virtually via telemedicine, the AVMA’s current policy is that veterinary telemedicine should only be conducted within an existing VCPR (38).

The last policy solution we examined was the introduction of a Veterinary Professional Associate (VPA) into the profession. We found the majority of technicians supported this solution but less than half of DVMs and practice managers/owners did. This may be the result of the nature of the arguments for and against this new type of professional: some technicians saw the VPA as a potential career pathway for them, while some DVMs were concerned about liability of having a VPA practice under their license and the introduction of the VPA increasing the DVM shortage. Our findings regarding technicians’ interest in the VPA concept is similar to Fults et al. (39), who found that the majority of 744 technicians they surveyed were moderately or extremely interested in the career path of becoming a VPA (or what they refer to as an Advanced Practice Registered Veterinary Nurse).

While less than half of the total veterinary professionals that we surveyed believed that a VPA would benefit the profession and their practice, more than half of our sample somewhat or strongly agreed that it would increase access to veterinary care for underserved populations. Additionally, pet owners had higher rates of comfort for a VPA performing urgent conditions and more invasive procedures (e.g., dental surgery, spaying, neutering, and treatment of urgent conditions) than a CVT. While there has been prior research on veterinary professionals’ perspectives of the VPA concept (26, 27), our results highlight the complex and diverse perspectives on this topic and thus the need for continued dialogue and deliberation among diverse stakeholders and professionals on the benefits and challenges to introducing this new type of professional.

Finally, our findings highlight the need for more focus on providing low-cost emergency care to pet owners. Among both pet owner samples, emergency care was the most common type of care pet owners were seeking when unable to access care. Furthermore, a new low-cost clinic that can provide sick and emergency care for pet owners was the resource rated as being most helpful among pet owners from both samples. While there are a growing number of programs that provide free or low-cost vaccines, spay and neuter, and other preventative care, programs for emergency care are less common. Our data suggest that this is a critical area for future work focused on enhancing access to care. One particular challenge of providing low-cost emergency care services is the overall high cost of running an emergency clinic due to the need for 24–7 staffing and the difficulty of recruiting and retaining veterinary professionals, especially given the high rates of burnout among veterinary emergency care providers (29). Thus, enhancing low-cost emergency services will likely require multi-pronged interventions including seed funding, programs (e.g., loan repayment, etc.) for increasing recruitment of veterinary professionals into emergency care, and training for practice managers/owners to increase workplace satisfaction and reduce burnout among professionals.

Our study has several limitations that should be considered in the interpretation of our results. First, while we recruited our sample from all registered veterinary professionals in the state of Colorado, our sample may not be fully representative of all veterinary professionals if there was response bias. Specifically, those who may have more personal experience with or interest in veterinary workforce and access to care challenges may have been more motivated to complete our survey. Furthermore, while our online sample of Colorado pet owners was generally representative of the overall state population in terms of demographics, previous studies have suggested that online samples recruited from panel providers, such as Qualtrics, may provide a biased sample, especially on animal-related issues (40). Future studies are needed on pet owner and veterinary professionals’ perspectives on access to care challenges and potential solutions using as representative of samples as possible.

## 5 Conclusion

Our findings suggest significant access to care challenges, reported from both the perspectives of veterinary professionals and pet owners in the state of Colorado. However, our findings also demonstrate support for a range of potential solutions to address these challenges, including expanded use of telemedicine, enhancing career pathways for and clarifying the role of veterinary technicians, grant and voucher programs for enhancing access to care, and expanding loan repayment programs to incorporate technicians and work in shelters or non-profit clinics. Additionally, our findings illustrate a strong need for more low-cost emergency clinics to expand access to care. To achieve these solutions, pet owners and veterinary professionals can conduct outreach and education to policymakers, professional associations, universities, and other stakeholders on these issues. Our results can be used in such outreach to demonstrate that both veterinary professionals and bonded families are in strong support of an array of programs and policies to enhance access to care and improve workforce challenges.

## Data availability statement

The raw data supporting the conclusions of this article upon request by the authors, without undue reservation.

## Ethics statement

The studies involving humans were approved by Colorado State University Institution Review Board Protocol #4775, titled “Veterinary Professional and Public Perceptions Towards Access to Care and Veterinary Workforce Challenges in Colorado”. The studies were conducted in accordance with the local legislation and institutional requirements. The participants provided their written informed consent to participate in this study.

## Author contributions

RN: Conceptualization, Data curation, Formal analysis, Funding acquisition, Investigation, Methodology, Project administration, Resources, Supervision, Validation, Writing – original draft, Writing – review & editing. VC: Data curation, Formal analysis, Investigation, Visualization, Writing – review & editing. DF: Conceptualization, Investigation, Methodology, Resources, Supervision, Writing – review & editing. AL: Investigation, Methodology, Writing – review & editing. AS: Conceptualization, Methodology, Supervision, Writing – review & editing. CV: Conceptualization, Methodology, Supervision, Writing – review & editing. LK: Supervision, Validation, Writing – original draft, Writing – review & editing. AM: Data curation, Formal analysis, writing – review and editing.
