# Supplementary material for: Veterinary and pet owner perspectives on addressing access to veterinary care and workforce challenges
Source: Front Vet Sci. 2024 Jul 4;11:1419295. doi: 10.3389/fvets.2024.1419295 (PMC11289980; doi:10.3389/fvets.2024.1419295)
Supplement: Supplementary file 1 [file Table_1.DOCX]

Supplementary Material

Below, we have provided a copy of the surveys provided to veterinary professionals and pet owners.

Veterinary Professional Survey on Access to Care and Veterinary Workforce Challenges

Start of Block: Intro questions- for all survey takers

Q1 Dear Participant,

We are conducting a research study on veterinary professionals’ attitudes and beliefs towards access to veterinary care and veterinary workforce challenges in Colorado. This study is a social science investigation on public attitudes towards a current event; the material presented in the survey is in no way representative of the beliefs of the research team. We would like you to take an online survey. Participation will take approximately 25-35 minutes. Your participation in this research is voluntary. If you decide to participate in the study, you may withdraw your consent and stop participation at any time without penalty.

We will be collecting data on your age, gender, type and area of practice, schooling, and information about your beliefs and attitudes towards access to care and workforce challenges in Colorado. We will also ask if you’d like to provide your email to participate in potential future interviews or focus groups. Providing your email is optional. When we report and share the results of the survey with others outside the research team, we will combine the data from all participants so that your individual responses will not be identifiable. We will keep your individual demographic data and responses to survey questions confidential; your data will be kept in a password-protected folder accessible only to the research team.

There is the potential for emotional distress related to some of the questions, as you will be asked questions related to veterinary workforce challenges and barriers to access to veterinary care. This discomfort is not expected to be any greater than anything you encounter in everyday life. If you decide to participate, you are free to stop at any time; you may also skip questions you do not want to answer. It is not possible to identify all potential risks in research procedures, but the researcher(s) have taken reasonable safeguards to minimize any known and potential (but unknown) risks.

To indicate your willingness to participate in this research and to continue on to the survey, please answer below. If you have any questions about the research, please contact Dr Rebecca Niemiec at rebecca.niemiec@colostate.edu. If you have any questions about your rights as a volunteer in this research, contact the CSU IRB at: csu_irb@colostate.edu ; 970-491-1553. The protocol number is 4775 and the protocol name is "Veterinary Professional and Public Perceptions Towards Access to Care and Veterinary Workforce Challenges in Colorado.”

- Yes, I am willing to participate (1)
- No I am not willing to participate (2)

Skip To: End of Survey If Dear Participant, We are conducting a research study on veterinary professionals’ attitudes and b... = No I am not willing to participate

| Page Break |  |
| --- | --- |

Q2 Are you a veterinarian or veterinary technician or are you a practice owner or manager in Colorado?

- Yes (1)
- No (2)

Skip To: End of Survey If Are you a veterinarian or veterinary technician or are you a practice owner or manager in Colorado? = No

Q159 How did you receive this survey link?

- Postcard mailing (1)
- Forwarded to me from friend/colleague (7)
- Email listserv (if so describe which listserv) (3) __________________________________________________
- Social media (4)
- Other (please describe) (6) __________________________________________________

Q3 What type of practice do you primarily work in?

- Companion animal only (1)
- Large animal only (2)
- Mixed animal (3)
- Shelter/nonprofit (4)
- Other (5) __________________________________________________

Display This Question:

If What type of practice do you primarily work in? = Companion animal only

Or What type of practice do you primarily work in? = Large animal only

Or What type of practice do you primarily work in? = Mixed animal

Q4 Do you work for a privately-owned, corporate-owned, or non-profit practice?

- Privately owned (not corporately owned) (1)
- Corporately owned (2)
- Non-profit (3)

Display This Question:

If What type of practice do you primarily work in? = Shelter/nonprofit

Q158 Does your shelter/nonprofit provide services for owned pets in your community?

- Yes (1)
- No (2)

Q9 Does your practice provide emergency services?

- Yes (1)
- No (2)

Q5 Which of the following best describes your current role? (check all that apply)

- Veterinarian (DVM/VMD) (1)
- Registered/certified veterinary technician or non-credentialed veterinary technician (2)
- Practice manager/leadership position (3)
- Practice owner (4)
- Other (6) __________________________________________________

| Page Break |  |
| --- | --- |

Q6 The focus of this survey is on understanding veterinary professional’s perceptions of the veterinary workforce and access to care challenges facing our communities. This survey was initiated through a collaborative effort between the Colorado Governor’s Office and the Animal-Human Policy Centerat Colorado State University. The design of the survey builds on research, including the 2018 Access to Veterinary Care Coalition (AVCC) report, suggesting that many households with pets are not able to get access to veterinary care. The 2018 AVCC report found that one out of four households in the US experienced barriers to receiving veterinary care. Further 86.7% of all respondents agreed that not being able to obtain needed veterinary care impacts the owner’s mental and emotional health.

 Additionally, a 2022 report by Mars Veterinary Healthfound that nearly 41,000 additional veterinarians will be needed to meet the needs of companion animal healthcare by 2030 and it is possible over 75 million pets in the U.S. may not have access to veterinary care by 2030 without intervention.

 This survey seeks to understand your perspective as a veterinary professional on these challenges and how they affect your practice and your community. The survey also asks about your perspective on a wide range of potential programs and policy solutions to address these access to care and workforce challenges. Your perspectives as a professional are critical to helping policy-makers, stakeholders, and others in developing solutions that can effectively address workforce challenges and increase access to veterinary care for underserved pets.

 In this survey, we use the following definitions from the AVCC 2018 report: ***Underserved populations*** of pets are pets with owners whose demographic, geographic, or economic characteristics impede or prevent access to veterinary care services. ***Access to veterinary care*** is recognizing when a pet needs care, having a veterinary service provider that is physically reachable, and being able to pay for the care. It includes wellness and preventative care, sick and emergency care. ***Owned pets*** are animals kept primarily for a person’s or family’s companionship, protection, and/or pleasure.

| Page Break |  |
| --- | --- |

End of Block: Intro questions- for all survey takers

Start of Block: For DVMS and practice managers only- clients and patient wait time

Q8 In the next few questions, we will ask about how quickly you can take on clients for preventative care, sick care or emergency care. According to the Access to Veterinary Care Coalition 2018 report: ***Preventative care*** is “services a pet [or livestock animal] receives to avoid illnesses, diseases, and behavioral problems. This includes such things as annual exams, shots or vaccinations; heartworm, flea, or tick preventions; and advice about how to care for a pet [or livestock animal] .” ***Sick care*** is defined as “treatment for an illness, physical injury, or behavioral problem that is NOT an emergency requiring immediate care.” ***Emergency care*** is “for an illness, physical injury, or behavioral problem that needs immediate treatment.”

Q11 What is your average lead time for seeing a ***new client*** for the following (skip this question if working in ER/specialty medicine only):

|  | Within 1 day (1) | Within 2 days (2) | Within 3 days (3) | Within a week (4) | Within 2 weeks (5) | Within 4 weeks (6) | More than a month (7) |
| --- | --- | --- | --- | --- | --- | --- | --- |
| Preventative care (1) |  |  |  |  |  |  |  |
| Sick care (2) |  |  |  |  |  |  |  |

Q10 What is your average lead time for seeing a ***current client*** for the following (skip this question if working in ER/specialty medicine only):

|  | Within 1 day (1) | Within 2 days (2) | Within 3 days (3) | Within a week (4) | Within 2 weeks (5) | Within 4 weeks (6) | More than a month (7) |
| --- | --- | --- | --- | --- | --- | --- | --- |
| Preventative care (1) |  |  |  |  |  |  |  |
| Sick care (2) |  |  |  |  |  |  |  |

Q13 Would you be able to take on additional clients at this time while being able to maintain a sustainable workload for you and your team?

- Yes (1)
- No (2)
- Not sure (describe) (3) __________________________________________________

Display This Question:

If Would you be able to take on additional clients at this time while being able to maintain a susta... = Yes

Q14 Overall, approximately how many new clients could you take in weekly over the next several months while being able to maintain a sustainable workload for you and your team?

- 0 (4)
- 1-5 (5)
- 5-10 (6)
- 10-20 (7)
- More/no limit (8)

End of Block: For DVMS and practice managers only- clients and patient wait time

Start of Block: For practice managers only- practice questions

| 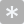 |
| --- |

Q128 How many full time equivalent (FTE) veterinarians does your practice currently employ?

________________________________________________________________

Q132 Are you currently advertising or interviewing to hire additional veterinarians?

- Yes (1)
- No (2)

| 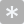 |
| --- |

Q133 How long on average does it currently take to fill a veterinarian position in your practice or place of work? (in months)

________________________________________________________________

| 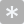 |
| --- |

Q7 On average, how many clients does your practice schedule per veterinarian per day?

________________________________________________________________

Q135 In the past year, on average, how often has your clinic had to divert clients because you can’t fit them into your schedule or address their condition in a reasonable time frame?

- Daily (1)
- Weekly (2)
- Monthly (3)
- Annually (4)
- Never (5)
- Don't know enough to estimate (6)

Q15 Do you believe your current patient load is too high, too low, or about right for maintaining financial success?

- Extremely low (1)
- Moderately low (2)
- Slightly low (3)
- Just right (4)
- Slightly high (5)
- Moderately high (6)
- Extremely high (7)

End of Block: For practice managers only- practice questions

Start of Block: For DVMs and techs only- patient load, econ barriers and euthanasia

Q12 In the past year, on average, how often has your clinic had to divert clients because you can’t fit them into your schedule or address their condition in a reasonable time frame?

- Daily (1)
- Weekly (2)
- Monthly (3)
- Annually (4)
- Never (5)
- Don't know enough to estimate (6)

Q16 Do you believe your current patient load is too high, too low, or about right for maintaining a sustainable work-life balance?

- Extremely low (1)
- Moderately low (2)
- Slightly low (3)
- Just right (4)
- Slightly high (5)
- Moderately high (6)
- Extremely high (7)

Display This Question:

If Do you believe your current patient load is too high, too low, or about right for maintaining a s... = Moderately high

Or Do you believe your current patient load is too high, too low, or about right for maintaining a s... = Extremely high

Or Do you believe your current patient load is too high, too low, or about right for maintaining a s... = Slightly high

Q17 To what extent does your patient load negatively influence your mental health?

- Not at all (1)
- Somewhat influences (2)
- Moderately influences (3)
- Influences a lot (4)
- Influences a great deal (5)

Display This Question:

If Do you believe your current patient load is too high, too low, or about right for maintaining a s... = Slightly high

Or Do you believe your current patient load is too high, too low, or about right for maintaining a s... = Moderately high

Or Do you believe your current patient load is too high, too low, or about right for maintaining a s... = Extremely high

Q18 To what extent does your patient load negatively influence your daily motivation to do your work?

- Not at all (1)
- Somewhat influences (2)
- Moderately influences (3)
- Influences a lot (4)
- Influences a great deal (5)

Q19 In the past year, on average, how often does your practice have to decline veterinary care for patients because the caretaker cannot afford to pay for the treatment?

- More than once a day (1)
- Once a day (2)
- Muliple times a week (3)
- Once a week (4)
- Multiple times a month (5)
- Once a month (6)
- Less than once a month (7)
- Never (8)
- I don't know enough to estimate (9)

Display This Question:

If In the past year, on average, how often does your practice have to decline veterinary care for pa... != Never

Q20 To what extent does not being able to provide treatment for sick patients for economic reasons impact your ***mental health***?

- Not at all (1)
- Somewhat influences (2)
- Moderately influences (3)
- Influences a lot (4)
- Influences a great deal (5)

Display This Question:

If In the past year, on average, how often does your practice have to decline veterinary care for pa... != Never

Q21 To what extent does not being able to provide treatment for sick patients for economic reasons impact your ***daily motivation to do your work***?

- Not at all (1)
- Somewhat influences (2)
- Moderately influences (3)
- Influences a lot (4)
- Influences a great deal (5)

Q23 In the past year, has your veterinary team had to euthanize an animal because the owner could not afford the treatment you recommended, and a different decision would have been made if the client had sufficient financial resources?

- Yes (1)
- No (2)

Display This Question:

If In the past year, has your veterinary team had to euthanize an animal because the owner could not... = Yes

Q24 To what extent do you believe that euthanizing an animal because the owner could not afford the treatment you recommended influences your ***mental health***?

- Not at all (1)
- Somewhat influences (2)
- Moderately influences (3)
- Influences a lot (4)
- Influences a great deal (5)

Display This Question:

If In the past year, has your veterinary team had to euthanize an animal because the owner could not... = Yes

Q25 To what extent does euthanizing an animal because the owner could not afford the treatment you recommended impact your ***daily motivation to do your work***?

- Not at all (1)
- Somewhat influences (2)
- Moderately influences (3)
- Influences a lot (4)
- Influences a great deal (5)

Q27 Overall, to what extent do you believe inadequate access to veterinary care is a problem in your area?

- Not at all a problem (1)
- A minor problem (2)
- A moderate problem (3)
- A significant problem (4)

End of Block: For DVMs and techs only- patient load, econ barriers and euthanasia

Start of Block: Shelter medicine only block

| 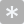 |
| --- |

Q31 How many full time equivalent (FTE) veterinarians are employed at your shelter?

________________________________________________________________

| 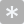 |
| --- |

Q32 How many full time equivalent (FTE) veterinary technicians are employed at your shelter?

________________________________________________________________

Q34 Has the number of full-time equivalent (FTE) veterinary staff at your shelter increased, decreased, or stayed the same over the past 3 years?

- Increased (1)
- Decreased (2)
- Stayed the same (3)
- Dont know (4)

Q29 What services do shelter-employed veterinarians provide for shelter-owned animals at your shelter? (check all that apply)

- Vaccines and parasite control (1)
- Spay/neuter (2)
- Infectious disease management (3)
- Dental procedures (4)
- Soft tissue surgery other that spay and neuter (5)
- Orthopedic procedures (6)
- Other (please describe): (7) __________________________________________________
- N/A (8)

Display This Question:

If Does your shelter/nonprofit provide services for owned pets in your community? = Yes

Q30 What services do shelter-employed veterinarians in your shelter provide for owned pets in your community? (check all that apply)

- Vaccinations (1)
- Parasite control (2)
- Spay/neuter (3)
- Acute care for sick and injured animals (4)
- Other (please describe): (5) __________________________________________________
- N/A (6)

Q35 Does your shelter use community veterinarians to perform procedures for shelter animals?

- Yes (1)
- No (2)

Display This Question:

If Does your shelter use community veterinarians to perform procedures for shelter animals? = Yes

Q36 Has the availability of community veterinarians for shelter animals changed in the last two years?

- No change in availability (1)
- More difficult for shelter animals to be seen by community veterinarians (2)
- Easier for shelter animals to be seen by community veterinarians (3)
- Don't know (4)

Q37 How long do animals at your shelter typically wait on a surgery list for spay and neuter surgeries?

- One day (1)
- 2-5 days (2)
- 1-3 weeks (3)
- More than a month (4)
- Don't know (5)

Q38 Do you believe your veterinary team at your shelter is currently understaffed?

- Yes (1)
- No (2)

Display This Question:

If Do you believe your veterinary team at your shelter is currently understaffed? = Yes

Q41 What do you think would be the ideal number of full time equivalent (FTE) veterinary staff to serve the needs of the animals at your shelter?

|  | 1 (1) | 2 (2) | 3 (3) | 4 (4) | 5+ (5) |
| --- | --- | --- | --- | --- | --- |
| Veterinarians (1) |  |  |  |  |  |
| Veterinary technicians (2) |  |  |  |  |  |

Display This Question:

If Do you believe your veterinary team at your shelter is currently understaffed? = Yes

Q39 How, if at all, does understaffing impact the services your shelter can provide to shelter animals?

________________________________________________________________

________________________________________________________________

________________________________________________________________

________________________________________________________________

________________________________________________________________

Display This Question:

If Do you believe your veterinary team at your shelter is currently understaffed? = Yes

Q40 How, if at all, does understaffing impact the services your shelter can provide to owned pets in the community?

________________________________________________________________

________________________________________________________________

________________________________________________________________

________________________________________________________________

________________________________________________________________

End of Block: Shelter medicine only block

Start of Block: For all- veterinary service provision for underserved populations

Q42 Veterinary service providers employ different strategies to address the needs of underserved populations of animals and people. For example, they may provide reduced fees and/or donated services, offer different payment options, offer financial support from a fund managed by the practice, or offer extended credit terms. What strategies have you and/or your practice or organization used in the past year to address the needs of underserved populations of animals and people?

________________________________________________________________

________________________________________________________________

________________________________________________________________

________________________________________________________________

________________________________________________________________

Q44 Consider a hypothetical grant program for clinics. The program could provide funds to private and non-profit clinics and organizations in your community to increase veterinary service for underserved populations of animals and people. The funds could be used in a variety of ways (e.g., angel funds, vouchers for owners to receive veterinary care, funding for mobile or low cost clinics or bringing veterinary services to the community, investing in telehealth, etc).

To what extent would you be interested in your practice or organization participating in such a grant program?

- Not at all interested (1)
- Somewhat interested (2)
- Moderately interested (3)
- Very interested (4)
- Extremely interested (5)

Q45 To what extent would you be interested in your practice or organization working with other private and non-profit clinics and organizations in your community to apply to such a grant program?

- Not at all interested (1)
- Somewhat interested (2)
- Moderately interested (3)
- Very interested (4)
- Extremely interested (5)

Q46 In an ideal world, how would you apply these grant funds to most effectively address the unique barriers to access to care in your community?

________________________________________________________________

________________________________________________________________

________________________________________________________________

________________________________________________________________

________________________________________________________________

Display This Question:

If Which of the following best describes your current role? (check all that apply) = Veterinarian (DVM/VMD)

Or Which of the following best describes your current role? (check all that apply) = Practice manager/leadership position

Or Which of the following best describes your current role? (check all that apply) = Practice owner

Q47 In Rhode Island, a program was founded in 2013 that provides the economic incentive of $125 vouchers to income-qualified pet owners. Income qualified pet owners would be approved through the program, rather than through veterinary clinics. The vouchers can be used by pet owners to reduce the costs of receiving veterinary services. If a similar program were implemented in your community for animal owners, do you think you or your practice would be willing to accept the vouchers as part of payment for services?

- Yes (1)
- No (2)
- Maybe (please describe) (3) __________________________________________________

Display This Question:

If In Rhode Island, a program was founded in 2013 that provides the economic incentive of $125 vouch... = Yes

Q136 Additionally, would you or your practice be able to subsidize a portion of the additional costs of care for voucher holders (e.g., waiving an exam fee)?

- Yes (1)
- No (2)

Display This Question:

If Additionally, would you or your practice be able to subsidize a portion of the additional costs o... = Yes

Q48 What percentage of the total cost of the visit would you consider subsidizing for voucher holders ?

- 10% of total service cost (1)
- 20% of total service cost (2)
- 50% of total service cost (3)
- Other (4) __________________________________________________

Display This Question:

If Additionally, would you or your practice be able to subsidize a portion of the additional costs o... = Yes

Q49 How many patients per month with vouchers would you be willing to provide subsidized services to?

- 1-5 (1)
- 5-10 (2)
- 10-15 (3)
- 15-20 (4)
- 20-30 (5)
- 30+ (6)

Q50 There has been discussion about the potential for implementing systems in communities to increase access to veterinary care for underserved populations of people and pets. This type of system recognizes that people’s animals are important, and when animals cannot get the care they need, they can suffer from illness or the risk of being surrendered to a shelter, which results in distress for the entire family. These systems seek to remove these family stressors by providing support to families and veterinary care for their animals. An example of such a is AlignCare [https://www.aligncarehealth.org/about], which connects pet families in need with veterinary service providers, community groups, and social service agencies. In AlignCare communities, veterinarians can sign up to be an AlignCare Veterinary Service Provider (VSP), so AlignCare families can choose a clinic to provide their pet with the services they need. For profit VSPs are asked to discount services by 20% for Aligncare families. Aligncare then covers 60% of the costs and the family provides 20% co-pay at the time services are rendered. Some services are never discounted, e.g., prescription foods, at the discretion of the VSP. Nonprofit VSPs are not asked to discount services, so in those instances, the family provides a 20% co-payment and AlignCare covers 80% of the costs. Veterinary Social Workers provide pet families with emotional support and coaching to help manage non-medical, pet-related issues. To what extent do you believe this type of system would be helpful in providing veterinary service to underserved populations of people and animals in your community?

- Not at all helpful (1)
- Somewhat helpful (2)
- Moderately helpful (3)
- Very helpful (4)

Display This Question:

If Which of the following best describes your current role? (check all that apply) = Veterinarian (DVM/VMD)

Or Which of the following best describes your current role? (check all that apply) = Practice manager/leadership position

Or Which of the following best describes your current role? (check all that apply) = Practice owner

Q51 If such a program existed in your community, would you be interested in participating by providing care for families in need in which the family pays a 20% copay when services are rendered, and AlignCare covers 60% or 80% of the costs (for profit vs non-profit, respectively)?

- Yes (1)
- Maybe (2)
- No (3)

Display This Question:

If If such a program existed in your community, would you be interested in participating by providin... = Yes

Q138 Please describe why you would be interested in participating.

________________________________________________________________

________________________________________________________________

________________________________________________________________

________________________________________________________________

________________________________________________________________

Display This Question:

If If such a program existed in your community, would you be interested in participating by providin... = Maybe

Or If such a program existed in your community, would you be interested in participating by providin... = No

Q139 Please describe why you wouldn't want to participate or are unsure about participating.

________________________________________________________________

________________________________________________________________

________________________________________________________________

________________________________________________________________

________________________________________________________________

End of Block: For all- veterinary service provision for underserved populations

Start of Block: For all- programs to help veterinary professionals enhance access to care

Q60 According to the 2018 Access to Veterinary Care Coalition report, many veterinarians would like to provide care to underserved pet populations but find it difficult to do so due to barriers such as time and workplace rules. The next few questions ask about your views on hypothetical incentive programs aimed at helping you and your clinic provide care to underserved pet populations.

Display This Question:

If Which of the following best describes your current role? (check all that apply) = Practice manager/leadership position

Or Which of the following best describes your current role? (check all that apply) = Practice owner

Q61 Some clinics in Colorado accept third party credit providers (e.g., Care Credit) that provide payment plans to clients with financial needs. But for some of these creditors, if the client doesn’t pay back their credit loans, the costs fall to the clinic. To what extent would a grant program backing these credit loans (i.e., to help cover the cost to clinics from defaulted loans) be useful in helping you and your clinic provide care to underserved pet populations?

- Not at all useful (1)
- Somewhat useful (2)
- Moderately useful (3)
- Very useful (4)

Display This Question:

If Which of the following best describes your current role? (check all that apply) = Practice manager/leadership position

Or Which of the following best describes your current role? (check all that apply) = Practice owner

Q62 To what extent would you be willing to provide interest free payment plans if they were backed by such a system?

- Not at all willing (1)
- Somewhat willing (2)
- Moderately willing (3)
- Very willing (4)

Q63 Knowing that there are federal and state loan forgiveness or repayment programs for veterinary professionals working in designated veterinary shortage areas, we are interested in your perspective on expanding potential loan forgiveness programs to include veterinary technicians and veterinary professionals working in low cost or shelter clinics.

 To what extent do you agree or disagree with the following statement: “The development of state-wide student loan repayment assistance programs for ***registered/certified veterinary technicians*** would increase access to veterinary care for underserved populations.”

- Strongly disagree (1)
- Somewhat disagree (2)
- Neither agree nor disagree (3)
- Somewhat agree (4)
- Strongly agree (5)

Q144 To what extent do you agree or disagree with the following statement: “The development of state-wide student loan repayment assistance programs for veterinary professionals who commit to working in ***low cost or shelter clinics*** for a period of time would increase access to veterinary care for underserved populations.”

- Strongly disagree (1)
- Somewhat disagree (2)
- Neither agree nor disagree (3)
- Somewhat agree (4)
- Strongly agree (5)

Display This Question:

If Which of the following best describes your current role? (check all that apply) = Veterinarian (DVM/VMD)

Q64 Knowing that there are federal loan forgiveness and repayment programs for working in designated veterinary shortage areas, how likely would you be (or would you have been at the beginning of your career) to work in the following settings to provide care to underserved pets if there was also a state-wide loan repayment assistance program for veterinary professionals who commit to working there for at least 3 years?

|  | Extremely unlikely (1) | Somewhat unlikely (2) | Neither likely nor unlikely (3) | Somewhat likely (4) | Extremely likely (5) |
| --- | --- | --- | --- | --- | --- |
| Low cost for-profit clinic (1) |  |  |  |  |  |
| Low cost non-profit clinic (2) |  |  |  |  |  |
| Shelter medicine (4) |  |  |  |  |  |

Display This Question:

If Which of the following best describes your current role? (check all that apply) = Registered/certified veterinary technician or non-credentialed veterinary technician

Q157 Knowing that there are federal loan forgiveness and repayment programs for veterinarians working in designated veterinary shortage areas, how likely would you be (or would you have been at the beginning of your career) to work in the following situations if the state-wide loan repayment assistance program were expanded to include ***registered/certified veterinary technicians*** who commit to working there for at least 3 years?

|  | Extremely unlikely (1) | Somewhat unlikely (2) | Neither likely nor unlikely (3) | Somewhat likely (4) | Extremely likely (5) |
| --- | --- | --- | --- | --- | --- |
| Low cost for-profit clinic (1) |  |  |  |  |  |
| Low cost non-profit clinic (2) |  |  |  |  |  |
| Shelter medicine (4) |  |  |  |  |  |

Q65 Do you volunteer or provide relief work for organizations that provide care to underserved areas/populations?

- Yes (If so, describe) (1) __________________________________________________
- No (2)

Q66 Do any of the following barriers prevent you from engaging in volunteer or relief work as often as you would like? (Check all that apply)

- Lack of time (1)
- Stress level too high (2)
- No economic incentives to do so (3)
- Don’t believe it makes an impact (4)
- Don’t know organizations to work with (5)
- Concerns about possible professional liability (6)
- Other (7) __________________________________________________

Q67 How frequently would you volunteer to provide preventative care to underserved areas/populations if there was a tax incentive to do so?

- Never (1)
- 1-2 times a year (2)
- 3-6 times a year (3)
- 6-9 times a year (4)
- >9 times a year (5)

End of Block: For all- programs to help veterinary professionals enhance access to care

Start of Block: For DVMS and managers- CVT/VTS questions

Q68 The following questions ask about your perspective about and experience working with veterinary technicians. Veterinary technicians became regulated by the State Board of Veterinary Medicine beginning January 2023 and must be registered according to Part 2 of the Veterinary Practice Act. Throughout 2023, certified veterinary technicians (CVTs) will be transitioning to registered veterinary technicians (RVTs), so both titles will be included in the questions below. Existing research on RVTs/CVTs has found high rates of attrition of veterinary technicians due to factors including not being able to work at the top of their knowledge and training, no career path for advancement, and insufficient compensation. Further, studies have found that retaining and recruiting technicians can increase a clinic’s efficiency, particularly when there is a 1:1 or greater ratio of veterinary technicians to veterinarians. We therefore want to hear about your experience working with veterinary technicians and your perspective on various proposed programs and policies for how the profession can better train, utilize, and retain technicians.

| 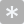 |
| --- |

Q69 How many full-time equivalent (FTE) registered/certified veterinary technicians (RVT/CVT) do you have in your clinic?

________________________________________________________________

| 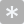 |
| --- |

Q70 How many full-time equivalent (FTE) registered/certified veterinary technicians (RVT/CVT) do you currently have per veterinarian in your clinic?

________________________________________________________________

| 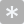 |
| --- |

Q71 What do you think would be the ideal number of veterinary RVTs/CVTs per veterinarian in your clinic to maximize efficiency and number of patients treated?

________________________________________________________________

Q72 How often do you believe you perform duties that RVTs/CVTs could perform?

- Never (1)
- Rarely (2)
- Sometimes (3)
- Often (4)

Q74 What tasks do you perform that you think your veterinary technicians could perform to make your practice more efficient?

________________________________________________________________

________________________________________________________________

________________________________________________________________

________________________________________________________________

________________________________________________________________

Q75 What currently prevents your technicians from performing these tasks?

________________________________________________________________

________________________________________________________________

________________________________________________________________

________________________________________________________________

________________________________________________________________

Q76 Some discussions have occurred about the potential for policy clarifying what tasks are appropriate for delegation under specific levels of supervision by veterinarians to CVTs/RVTs. How helpful would this be for you to more efficiently work with the veterinary technicians in your practice?

- A hinderance (1)
- Not at all helpful (2)
- Somewhat helpful (3)
- Moderately helpful (4)
- Very helpful (5)

Q77 In 2020, the Community College of Denver (CCD) announced a new Veterinary Technology U.S. Department of Labor Registered Apprenticeship Program, in which student apprentices work part time receiving structured on-the-job training at clinics in addition to hours spent in school to receive an Associate’s Degree. How willing would you be to hire a part-time veterinary technology student apprentice through a similar program?

- Not at all willing (1)
- Somewhat willing (2)
- Moderately willing (3)
- Very willing (4)

Q78 Would your willingness to hire this student increase if offered a grant to help cover the costs of paying the student’s hourly rate?

- Not at all (1)
- Slighlty increase (2)
- Moderately increase (3)
- Greatly increase (4)

Q161 Why would you be willing or unwilling to hire a student through this program?

________________________________________________________________

________________________________________________________________

________________________________________________________________

________________________________________________________________

________________________________________________________________

Q162 How confident do you feel in assessing the capabilities, skills, knowledge, etc. of the veterinary technicians/specialists in your clinic?

- Not at all confident (1)
- Somewhat confident (2)
- Moderately confident (3)
- Very confident (4)

Q80 Would you take a CE course on leadership development that covers assessing the capabilities, skills, and knowledge of RVTs/CVTs and applying that assessment to fully utilize the demonstrated, tested, competence of RVTs/CVTs?

- Yes (1)
- No (2)

Q163 Why would you or wouldn't you take this course?

________________________________________________________________

Q81 The following questions ask about your perspective on Veterinary Technician Specialists (VTSs). VTSs are credentialed veterinary technicians who have completed extra training in a specialty through a technician specialty academy and have passed a certifying exam. In general, VTS candidates must have worked as a credentialed veterinary technician for a minimum of three to five years.

Q82 How knowledgeable are you about the Veterinary Technician Specialist (VTS) credential?

- Not knowledgeable at all (1)
- Slightly knowledgeable (2)
- Moderately knowledgeable (3)
- Very knowledgeable (4)

Q83 Do you currently employ any Veterinary Technician Specialists (VTS’s)?

- Yes (1)
- No (2)

Q84 What influences your decision-making on whether or not to hire Veterinary Technician Specialists over RVTs/CVTs without this designation?

________________________________________________________________

________________________________________________________________

________________________________________________________________

________________________________________________________________

________________________________________________________________

Q85 There have been some conversations around expanding the roles of RVTs/CVTs, particularly those with the VTS designation. A gap in existing knowledge around this topic is to what extent veterinarians are performing tasks that could be productively delegated to veterinary technicians. Veterinary professionals and stakeholders may have different perspectives on this idea of expanding the roles of RVTs/CVTs/VTS's, so we'd like to better understand your perspective on this topic. Specifically, we would like to know your comfort level around RVTs/CVTs or VTS’s performing the following tasks, to inform conversations on whether they should become permissible in general practices under federal and state guidelines. For each task listed below, check if you would support an RVT/CVT and/or a specialty trained VTS completing the task if it is in their specialty area and under the supervision of a veterinarian. If you don't support either completing the task, don't check either box next to the task:

|  | RVT/CVT (1) | VTS (2) |
| --- | --- | --- |
| Preventive medicine care (vaccines, parasite control) (1) |  |  |
| Establish a veterinary-client-patient relationship (2) |  |  |
| Leadership development of veterinary teams (3) |  |  |
| Physical examinations, understanding when escalation to a DVM is needed (4) |  |  |
| Develop differential diagnoses and diagnostic plans, and understand when escalation to a DVM is needed (5) |  |  |
| Interpret laboratory and radiographic results, and understand when escalation to a DVM is needed (6) |  |  |
| Prescribe medication as allowed by Federal regulations, understanding when escalation to a DVM is needed (7) |  |  |
| End of life counseling and euthanasia (8) |  |  |
| Surgical procedures external to body cavity (9) |  |  |
| Spays for owned animals (10) |  |  |
| Spays for animals in shelter situations (11) |  |  |
| Develop protocols for biological risk management (12) |  |  |
| Provide tele-triage (13) |  |  |
| Provide telemedicine, understanding when escalation to a DVM is needed (14) |  |  |
| Dental procedures including single root tooth extractions and suturing of gingiva (15) |  |  |
| Dental procedures including multiple root tooth extractions and suturing of gingiva (16) |  |  |
| Coordinate case management between primary and referral veterinarian (17) |  |  |
| Diagnose and treat infections of the ears, eyes and skin, knowing when to refer/escalate to a DVM (18) |  |  |

Q86 Are there other specific types of procedures that you would support a VTS being trained in and practicing? If so, describe:

________________________________________________________________

________________________________________________________________

________________________________________________________________

________________________________________________________________

________________________________________________________________

| Page Break |  |
| --- | --- |

Q87 Please indicate your level of agreement with the following statements. If you don’t know enough about VTS’s to answer, please leave it blank:

|  | Strongly disagree (1) | Somewhat disagree (2) | Neither agree nor disagree (3) | Somewhat agree (4) | Strongly agree (5) |
| --- | --- | --- | --- | --- | --- |
| RVTs/CVTs are difficult to find. (1) |  |  |  |  |  |
| It is difficult to keep RVTs/CVTs employed in my practice over time (2) |  |  |  |  |  |
| Veterinary Technician Specialists are difficult to find. (3) |  |  |  |  |  |
| It is difficult to keep Veterinary Technician Specialists employed in my practice over time (4) |  |  |  |  |  |
| Veterinary Technician Specialists can perform more types of tasks at a higher quality than RVTs/CVTs without the specialist designation. (5) |  |  |  |  |  |
| I would hire a Veterinary Technician Specialist (VTS) over a technician without the specialist designation if more VTS’s were available. (6) |  |  |  |  |  |
| I would offer a higher salary for VTS’s compared to veterinary technicians without the specialist designation. (7) |  |  |  |  |  |
| If more CVTs obtained a VTS designation, this would increase access to veterinary care for underserved populations (8) |  |  |  |  |  |

Q88 Do you think more CVTs obtaining a VTS designation would....

|  | Yes (1) | No (2) | Not sure (3) |
| --- | --- | --- | --- |
| positively benefit the profession? (1) |  |  |  |
| positively benefit your practice? (2) |  |  |  |

Q89 To what extent do you think the following would help increase the number of CVTs receiving a VTS designation?

|  | Not at all (1) | Slightly increase (2) | Moderately increase (3) | Greatly increase (4) |
| --- | --- | --- | --- | --- |
| More clear role delineation of the role of VTSs (1) |  |  |  |  |
| More structured support (resident programs, mentorships) aimed at helping technicians obtain a VTS designation (2) |  |  |  |  |
| Grant funds available to cover the cost of getting the designation (3) |  |  |  |  |

Q90 What other ideas do you have for increasing the number of CVTs pursuing a VTS designation?

________________________________________________________________

________________________________________________________________

________________________________________________________________

________________________________________________________________

________________________________________________________________

End of Block: For DVMS and managers- CVT/VTS questions

Start of Block: For DVMs and techs- Contextualized/Incremental care

Q53 Skipper et al. (2021) define ***“contextualized care”*** as a case management strategy that acknowledges that “different treatment modalities may be equally valid in different contexts. The most appropriate pathway for each patient and owner should be navigated through an iterative process of shared decision-making; we cannot separate clinic decisions from their social contexts.” This concept of contextualized care aligns closely with the concept of ***incremental veterinary care***, defined by the program for pet health equity as “a case management strategy that utilizes the intuitive judgment of the veterinarian to develop a tiered diagnostic and dynamic therapeutic options over time. Non-critical procedures are avoided to help control costs. It relies on the clinical judgment of the veterinarian, active follow-up of case progression, and, when appropriate, in-home care that can be provided by the client.” In the following questions, we would like to know more about your perspective on implementing both contextualized and incremental care.

Q54 To what extent do you agree or disagree with the following statement: “More widespread implementation of contextualized and incremental veterinary care in clinics would increase access to veterinary care for underserved populations.”

- Strongly disagree (1)
- Somewhat disagree (2)
- Neither agree nor disagree (3)
- Somewhat agree (4)
- Strongly agree (5)

Q55 To what extent do you agree or disagree with the following statement: "I feel confident offering contextualized or incremental veterinary care for a given condition.”

- Strongly disagree (1)
- Somewhat disagree (2)
- Neither agree nor disagree (3)
- Somewhat agree (4)
- Strongly agree (5)

Q142 To what extent do you agree or disagree with the following statement: "I feel confident communicating with clients about the relative impact and cost of options along a spectrum of care."

- Strongly disagree (1)
- Somewhat disagree (2)
- Neither agree nor disagree (3)
- Somewhat agree (4)
- Strongly agree (5)

Q58 What concerns do you have about offering contextualized or incremental care to your clients?

________________________________________________________________

Q59 What resources would help you better offer contextualized or incremental care to your clients?

________________________________________________________________

________________________________________________________________

________________________________________________________________

________________________________________________________________

________________________________________________________________

Q143 Would you take a continuing education (CE) course on implementing and communicating contextualized care to your clients?

- Yes (1)
- No (2)

End of Block: For DVMs and techs- Contextualized/Incremental care

Start of Block: For techs- CVT/VTS questions

Q95 The below questions ask about your experience as a veterinary technician. Veterinary technicians became regulated by the State Board of Veterinary Medicine beginning January 2023 and must be registered pursuant to Part 2 of the Veterinary Practice Act. Throughout 2023, certified veterinary technicians (CVTs) will be transitioning to registered veterinary technicians (RVTs), so both titles will be included in the questions below. Existing research on RVTs/CVTs has found high rates of attrition of veterinary technicians due to factors including not being able to work at the top of their knowledge and training, no career path for advancement, and insufficient compensation. Further, studies have found that retaining and recruiting technicians can increase a clinic’s efficiency, particularly when there is a 1:1 or greater ratio of veterinary technicians to veterinarians. We therefore want to hear about your experience as a veterinary technician and your perspective on various proposed programs and policies for how the profession can better train, utilize, and retain technicians.

| 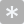 |
| --- |

Q153 What do you think would be the ideal number of veterinary RVTs/CVTs per veterinarian in your clinic to maximize efficiency and number of patients treated?

________________________________________________________________

Q92 Are there tasks that DVMs perform that you believe you could perform to make your practice more efficient? Please describe below.

________________________________________________________________

________________________________________________________________

________________________________________________________________

________________________________________________________________

________________________________________________________________

Q94 What currently prevents you from performing these tasks?

________________________________________________________________

________________________________________________________________

________________________________________________________________

________________________________________________________________

________________________________________________________________

Q146 Some discussions have occurred about the potential for policy clarifying what tasks are appropriate for delegation under specific levels of supervision by veterinarians to CVTs/RVTs. How helpful would this be for you to more efficiently work with the veterinarians in your practice?

- A hinderance (1)
- Not at all helpful (2)
- Somewhat helpful (3)
- Moderately helpful (4)
- Very helpful (5)

Q164 Would you take a course on how to work with veterinarians in your clinic to efficiently and effectively apply your skills as a veterinary technician?

- Yes (1)
- No (2)

Q165 Why/why not?

________________________________________________________________

Q91 The following questions ask about your perspective on Veterinary Technician Specialists (VTS’s). VTSs are credentialed veterinary technicians who have completed extra training in a specialty through a technician specialty academy and have passed a certifying exam. In general, VTS candidates must have worked as a credentialed veterinary technician for a minimum of three to five years.

Q97 Do you have a VTS credential?

- Yes (1)
- No (2)

Display This Question:

If Do you have a VTS credential? = Yes

Q152 Please describe whether this has led to financial or other benefits in your career.

________________________________________________________________

________________________________________________________________

________________________________________________________________

________________________________________________________________

________________________________________________________________

Display This Question:

If Do you have a VTS credential? = No

Q96 How knowledgeable are you about the Veterinary Technician Specialist (VTS) credential?

- Not knowledgeable at all (1)
- Slightly knowledgeable (2)
- Moderately knowledgeable (3)
- Very knowledgeable (4)

Q147 There have been some conversations around expanding the roles of RVTs/CVTs, particularly those with the VTS designation. A gap in existing knowledge around this topic is to what extent veterinarians are performing tasks that could be productively delegated to veterinary technicians. Veterinary professionals and stakeholders may have different perspectives on this idea of expanding the roles of RVTs/CVTs/VTS's, so we'd like to better understand your perspective on this topic. Specifically, we would like to know your comfort level around RVTs/CVTs or VTS’s performing the following tasks, to inform conversations on whether they should become permissible under federal and state guidelines. For each task listed below, check if you would support an RVT/CVT and/or a specialty trained VTS completing the task if it is in their specialty area and under the supervision of a veterinarian. If you don't support either completing the task, don't check either box next to the task:

|  | RVTs/CVTs (1) | VTS's (2) |
| --- | --- | --- |
| Preventive medicine care (vaccines, parasite control) (1) |  |  |
| Establish a veterinary-client-patient relationship (2) |  |  |
| Leadership development of veterinary teams (3) |  |  |
| Physical examinations, understanding when escalation to a DVM is needed (4) |  |  |
| Develop differential diagnoses and diagnostic plans, and understand when escalation to a DVM is needed (5) |  |  |
| Interpret laboratory and radiographic results, and understand when escalation to a DVM is needed (6) |  |  |
| Prescribe medication as allowed by Federal regulations, understanding when escalation to a DVM is needed (7) |  |  |
| End of life counseling and euthanasia (8) |  |  |
| Surgical procedures external to body cavity (9) |  |  |
| Spays for owned animals (10) |  |  |
| Spays for animals in shelter situations (11) |  |  |
| Develop protocols for biological risk management (12) |  |  |
| Provide tele-triage (13) |  |  |
| Provide telemedicine, understanding when escalation to a DVM is needed (14) |  |  |
| Dental procedures including single root tooth extractions and suturing of gingiva (15) |  |  |
| Dental procedures including multiple root tooth extractions and suturing of gingiva (16) |  |  |
| Coordinate case management between primary and referral veterinarian (17) |  |  |
| Diagnose and treat infections of the ears, eyes and skin, knowing when to refer/escalate to a DVM (18) |  |  |

Q151 Are there other specific types of procedures that you would support a VTS being trained in and practicing? If so, describe:

________________________________________________________________

________________________________________________________________

________________________________________________________________

________________________________________________________________

________________________________________________________________

Q149 To what extent do you agree/disagree with the following statement: "If more CVTs obtained a VTS designation, this would increase access to veterinary care for underserved populations."

- Strongly disagree (1)
- Somewhat disagree (2)
- Neither agree nor disagree (3)
- Somewhat agree (4)
- Strongly agree (5)

Q150 Do you think more CVTs obtaining a VTS designation would....

|  | Yes (1) | No (2) | Not sure (3) |
| --- | --- | --- | --- |
| positively benefit the profession? (1) |  |  |  |
| would positively benefit your practice? (2) |  |  |  |

Q98 To what extent do you think the following would help increase the number of CVTs receiving a VTS designation?

|  | Not at all (1) | Slightly increase (2) | Moderately increase (3) | Greatly increase (4) |
| --- | --- | --- | --- | --- |
| More clear role delineation of the role of VTSs (1) |  |  |  |  |
| More structured support (resident programs, mentorships) aimed at helping technicians obtain a VTS designation (2) |  |  |  |  |
| Grant funds available to cover the cost of getting the designation (3) |  |  |  |  |

Q148 What other ideas do you have for increasing the number of CVTs pursuing a VTS designation?

________________________________________________________________

________________________________________________________________

________________________________________________________________

________________________________________________________________

________________________________________________________________

End of Block: For techs- CVT/VTS questions

Start of Block: For all- Midlevel practitioner

Q100 In the next few questions, we would like to know your perspective on the potential introduction of a mid-level practitioner, or veterinary professional associate (VPA) into the profession. There has been an effort to develop a Masters of Veterinary Clinical Care (MSB-VCC) degree program to train such veterinary professional associates. Graduates of a program like this would be trained in clinical case management and would work under the supervision of a veterinarian, who determines the level of appropriate delegation.

Q101 To what extent do you agree/disagree with the following statement: “The development of a “mid-level” veterinary professional associate (VPA) through a Masters of Veterinary Clinical Care (MSB-VCC) degree would increase access to veterinary care for underserved populations.”

- Strongly disagree (1)
- Somewhat disagree (2)
- Neither agree nor disagree (3)
- Somewhat agree (4)
- Strongly agree (5)

Q154 A gap in existing knowledge around the topic of a mid-level professional is to what extent veterinarians are performing tasks that they believe could be productively delegated to mid-level professionals. Veterinary professionals and stakeholders may have different perspectives on this idea of delegating roles to a potential mid-level practitioner, so we'd like to better understand your perspective on this topic. Specifically, we would like to know your comfort level around mid-level practitioners performing the following tasks, to inform conversations on whether they should become permissible under federal and state guidelines. For each task listed below, check it if you would support a mid-level practitioner completing the task under the supervision of a veterinarian. If you don't support a mid-level practitioner completing the task, don't check the box next to the task.

- Preventive medicine care (vaccines, parasite control) (1)
- Establish a veterinary-client-patient relationship (2)
- Leadership development of veterinary teams (3)
- Physical examinations, understanding when escalation to a DVM is needed (4)
- Develop differential diagnoses and diagnostic plans, and understand when escalation to a DVM is needed (5)
- Interpret laboratory and radiographic results, and understand when escalation to a DVM is needed (6)
- Prescribe medication as allowed by Federal regulations, understanding when escalation to a DVM is needed (7)
- End of life counseling and euthanasia (8)
- Surgical procedures external to body cavity (9)
- Spays for owned animals (10)
- Spays for animals in shelter situations (11)
- Develop protocols for biological risk management (12)
- Provide tele-triage (13)
- Provide telemedicine, understanding when escalation to a DVM is needed (14)
- Dental procedures including single root tooth extractions and suturing of gingiva (15)
- Dental procedures including multiple root tooth extractions and suturing of gingiva (16)
- Coordinate case management between primary and referral veterinarian (17)
- Diagnose and treat infections of the ears, eyes and skin, knowing when to refer/escalate to a DVM (18)

Q103 Are there other specific types of procedures that you would support a “mid-level” veterinary professional associate (VPA) being trained in and practicing? If so, describe:

________________________________________________________________

________________________________________________________________

________________________________________________________________

________________________________________________________________

________________________________________________________________

Q104 Are there tasks that you engage in as a veterinarian in your clinic or organization that you think a trained “mid-level” veterinary professional associate (VPA) could complete instead but a veterinary technician or VTS could not?

- Yes (1)
- No (2)
- Not sure (3)

Display This Question:

If Are there tasks that you engage in as a veterinarian in your clinic or organization that you thin... = Yes

Q105 If so, what tasks? Please list below.

________________________________________________________________

Q107 Overall, do you think a veterinary professional associate (VPA) would positively benefit the profession?

- Yes (1)
- No (2)
- Not sure (3)

Q108 Overall, do you think a veterinary professional associate (VPA) would positively benefit your practice?

- Yes (1)
- No (2)
- Not sure (3)

Display This Question:

If Which of the following best describes your current role? (check all that apply) = Veterinarian (DVM/VMD)

Or Which of the following best describes your current role? (check all that apply) = Practice manager/leadership position

Or Which of the following best describes your current role? (check all that apply) = Practice owner

Q109 Would you hire a VPA in your practice?

- Yes (1)
- No (2)

Q110 Below, describe why you answered "yes" or "no" in the above questions.

________________________________________________________________

End of Block: For all- Midlevel practitioner

Start of Block: For all- Telemedicine

Q111 In this survey, we use the AVMA’s definition of telemedicine and telehealth. Veterinary ***telehealth*** includes: teleadvice, tele-education, telemonitoring, telesupervision, and teletriage to help veterinarians deliver veterinary education virtually. Veterinary ***telemedicine*** is the remote practice of veterinary medicine through the use of telehealth technologies that allows a veterinarian to treat an individual patient virtually.

Q112 To what extent do you agree/disagree with the following statement: “Expanding the use of telemedicine would increase access to veterinary care for underserved populations.”

- Strongly disagree (1)
- Somewhat disagree (2)
- Neither agree nor disagree (3)
- Somewhat agree (4)
- Strongly agree (5)

Q114 How often does your practice use veterinary telemedicine?

- Multiple times a day (1)
- Once a day (2)
- Multiple times a week (3)
- Once a week (4)
- Multiple times a month (5)
- Once a month (6)
- Less often (7)
- Never (8)

Q115 Which of the following veterinary services does your practice primarily use telemedicine for? (Check all that apply)

- Follow-up (2)
- Triage (3)
- After hours calls (4)
- Pre-surgery visit (5)
- Post-surgery visit (6)
- Client education (7)
- Other (8) __________________________________________________

| Page Break |  |
| --- | --- |

Q116 Are there veterinary services that you engage in as a veterinarian in your clinic or organization that are only being performed in person that you think could be performed via telemedicine? If so, describe what tasks below.

________________________________________________________________

Q117 Which of the following barriers prevent full implementation of telemedicine into your practice? (Check all that apply)

- Cost (1)
- Technology (2)
- Confusion over how to implement/charge for telemedicine (3)
- Acceptance and training of personnel (4)
- Client interest (5)
- Decreased in-person caseload (6)
- Revenue loss (7)
- Other (8) __________________________________________________

Q118 In May 2023 Senate Bill 1053 was signed into law in Arizona which allows veterinarians licensed in Arizona to establish a veterinarian-client-patient relationship (VCPR) through telemedicine. Veterinary professionals and stakeholders may have different perspectives on this idea of establishing a VCPR through telemedicine, so we’d like to better understand your perspective on this topic. To what extent do you think a similar law in Colorado would positively or negatively impact the profession?

- Strong negative impact (1)
- Moderate negative impact (2)
- Slight negative impact (5)
- No negative or positive impact (6)
- Slight positive impact (3)
- Moderate positive impact (4)
- Strong positive impact (7)

Q119 To what extent do you agree/disagree with the following statement: “The ability to establish a virtual veterinarian-client-patient relationship (VCPR) through telemedicine would increase the amount of care that veterinary professionals could provide to underserved populations.”

- Strongly disagree (1)
- Somewhat disagree (2)
- Neither agree nor disagree (3)
- Somewhat agree (4)
- Strongly agree (5)

Q120 Are there any resources that would help you better integrate telemedicine in your practice?

________________________________________________________________

End of Block: For all- Telemedicine

Start of Block: For all- Final Demographics

| 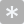 |
| --- |

Q121 How long have you been in your current role? (years)

________________________________________________________________

Q122 In what type of area do you practice?

- Urban (1)
- Suburban (2)
- Rural (3)

Q123 In which county do you practice?

▼ Adams (1) ... Yuma (64)

| 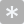 |
| --- |

Q124 What year did you graduate veterinary school or veterinary technology school (if applicable)?

________________________________________________________________

Display This Question:

If Which of the following best describes your current role? (check all that apply) = Registered/certified veterinary technician or non-credentialed veterinary technician

Q155 Are you currently certified or registered?

- Yes (1)
- No (2)

Display This Question:

If Are you currently certified or registered? = Yes

Q125 What year did you become certified or registered?

________________________________________________________________

Q156 Is there anything else you’d like to share related to the topics of veterinary workforce challenges and access to care?

________________________________________________________________

________________________________________________________________

________________________________________________________________

________________________________________________________________

________________________________________________________________

| 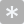 |
| --- |

Q126 Thank you so much for taking the time to complete this survey. Your thoughtful response is critical to informing state-wide policy discussions on addressing access to care and veterinary workforce challenges. If you'd like to be contacted about potential future focus groups or interviews on this topic, please provide your email below.

________________________________________________________________

End of Block: For all- Final Demographics

Colorado Public Access to Veterinary Care Survey

Start of Block: Default Question Block

Q1 Dear Participant,

 We are conducting a research study on public attitudes and beliefs towards access to veterinary care in Colorado. This study is a social science investigation on public attitudes and behaviors related to obtaining veterinary care; the material presented in the survey is in no way representative of the beliefs of the research team. We would like you to take an online survey. Participation will take approximately 15 minutes. Your participation in this research is voluntary. If you decide to participate in the study, you may withdraw your consent and stop participation at any time without penalty. We will be collecting data on your age, gender, area of residence, and information about your beliefs and attitudes and behaviors towards veterinary care. When we report and share the results of the survey with others outside the research team, we will combine the data from all participants so that your individual responses will not be identifiable. We will keep your individual demographic data and responses to survey questions confidential; your data will be kept in a password-protected folder accessible only to the research team.

 There is the potential for emotional distress related to some of the questions, as you will be asked questions related to challenges to trying to access veterinary care. This discomfort is not expected to be any greater than anything you encounter in everyday life. If you decide to participate, you are free to stop at any time; you may also skip questions you do not want to answer. It is not possible to identify all potential risks in research procedures, but the researcher(s) have taken reasonable safeguards to minimize any known and potential (but unknown) risks. You will be paid an amount determined through Qualtrics Survey Software for completing the survey. Qualtrics will be fully responsible for determining pay rates and administering payment.

 ***To indicate your willingness to participate in this research and to continue on to the survey, please answer below.*** If you have any questions about the research, please contact Dr Rebecca Niemiec at rebecca.niemiec@colostate.edu. If you have any questions about your rights as a volunteer in this research, contact the CSU IRB at: csu_irb@colostate.edu; 970-491-1553. The protocol number is 4775 and the protocol name is "Veterinary Professional and Public Perceptions Towards Access to Care and Veterinary Workforce Challenges in Colorado.”

- Yes, I am willing to participate in this research study (1)
- No I am not willing to participate in this research study (2)

Skip To: End of Block If Dear Participant, We are conducting a research study on public attitudes and beliefs towards acce... = No I am not willing to participate in this research study

Q2 This study is by Colorado State University’s Center for Animal-Human Policy about Colorado pet owners. The purpose of this survey is to understand pet owners’ experiences getting the care they want for their pet and any problems they may have in getting this care. There are no right or wrong answers. For the purposes of this survey, we are defining a pet owner as someone who has a dog or cat. Do you have a dog or a cat now or have you had a dog or cat in the past two years?

- Yes, I have a dog or cat now (1)
- Yes I don’t have a dog or cat now but have had a dog or cat in the past 2 years (2)
- No (3)

Skip To: End of Block If This study is by Colorado State University’s Center for Animal-Human Policy about Colorado pet ow... = No

End of Block: Default Question Block

Start of Block: Demographics

| 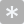 |
| --- |

Q40 What zip code do you live in?

________________________________________________________________

| Page Break |  |
| --- | --- |

Q54 What is your age?

- 18-34 (1)
- 35-54 (2)
- 55 and over (3)

Q123 In which county do you live?

▼ Adams (1) ... Yuma (64)

| Page Break |  |
| --- | --- |

Q46 Which of the best describes where you live?

- Large city (over 100,000 people) (1)
- Small city (between 20,000 and 100,000 people) (2)
- Town (between 5,000 and 20,000 people) (3)
- Small town (fewer than 5,000 people) (4)
- Rural area- non farm (5)
- Rural area-farm (6)
- Not sure (7)

Q47 Do you live in a house, apartment, condo, duplex, mobile home, or some other type of dwelling?

- House (1)
- Apartment (2)
- Condo (3)
- Duplex (4)
- Mobile home (5)
- Other (6) __________________________________________________

| Page Break |  |
| --- | --- |

Q48 Are you of Hispanic, Latino/a, or Spanish Origin?

- Yes (1)
- No (2)
- Not sure (3)

Q49 How do you describe your race? [Check all that apply]

- American Indian or Alaskan Native (1)
- Asian (2)
- Black or African American (3)
- Native Hawaiian or Other Pacific Islander (4)
- White (5)
- Other (6) __________________________________________________
- Rather not say (7)

| Page Break |  |
| --- | --- |

Q50 What is the highest grade of year of school you completed?

- 11th grade or less (1)
- High school graduate or GED (2)
- 1-3 years of college or technical school (3)
- Bachelor’s degree (4)
- Graduate or Professional Degree degree (5)

Q51 Thinking of all the income everyone in your household gets, such as work, retirement, Social Security, and other money from the government. If you add together everyone’s before-tax income for the year, would it be?

- Less than $50,000 (1)
- $50,000-$100,000 (2)
- Over $100,000 (3)

| Page Break |  |
| --- | --- |

Q52 With which gender do you most identify?

- Woman (1)
- Man (2)
- Transgender Woman (3)
- Transgender Man (4)
- Nonbinary/Non-conforming (5)
- Not listed (6)
- Prefer not to answer (7)

Q53 Did you or anyone in your family receive government benefits/assistance (SNAP, Medicaid, unemployment insurance, housing assistance, SSI, SSDI, etc.) in the last year?

- Yes (1)
- No (2)

End of Block: Demographics

Start of Block: Basic pet history

Q3 First, we would like to learn more about your pets. How many dogs do you own now?

▼ 0 (1) ... 5 or more (6)

Q4 How many cats do you own or regularly care for now?

▼ 0 (1) ... 5 or more (6)

Q5 How many dogs have you owned in the past two years that you do not have anymore?

▼ 0 (1) ... 5 or more (6)

Q6 How many cats have you owned or regularly cared for in the past two years that you do not have anymore?

▼ 0 (1) ... 5 or more (6)

| Page Break |  |
| --- | --- |

Q7 If you have dogs, how old are the dog(s) you have now? (Your best guess of age is fine)

|  | Age (years) |
| --- | --- |

|  | 0 | 2 | 4 | 6 | 8 | 10 | 12 | 14 | 16 | 18 | 20 |
| --- | --- | --- | --- | --- | --- | --- | --- | --- | --- | --- | --- |

| Dog #1 () | 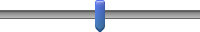 |
| --- | --- |
| Dog #2 () | 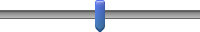 |
| Dog #3 () | 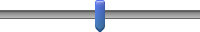 |
| Dog #4 () | 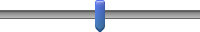 |
| Dog #5 () | 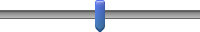 |

Q53 If you have cats, how old are the cat(s) you have now? (Your best guess of age is fine)

|  | Age (years) |
| --- | --- |

|  | 0 | 2 | 4 | 6 | 8 | 10 | 12 | 14 | 16 | 18 | 20 |
| --- | --- | --- | --- | --- | --- | --- | --- | --- | --- | --- | --- |

| Cat #1 () | 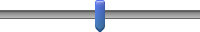 |
| --- | --- |
| Cat #2 () | 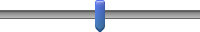 |
| Cat #3 () | 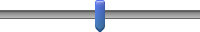 |
| Cat #4 () | 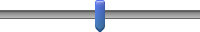 |
| Cat #5 () | 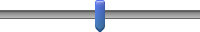 |

| Page Break |  |
| --- | --- |

Q8 Where did you get your pets from? [check all that apply]

- Adopted from an animal shelter (1)
- Adopted from a foster-based animal rescue organization (2)
- Bought from a pet store (3)
- Bought from a breeder (4)
- Received from a friend or family member (5)
- Was a stray that I took in (6)
- My pet had babies (10)
- Internet (7)
- Unsure (8)
- Other: (9) __________________________________________________

| Page Break |  |
| --- | --- |

Q9 We'd like to know about the breeding status of your pets. Which of the following statements about your pets is true?

- All my pets are spayed or neutered (1)
- Some of my pets are spayed or neutered (2)
- None of my pets are spayed or neutered (3)

Display This Question:

If We'd like to know about the breeding status of your pets. Which of the following statements about... = All my pets are spayed or neutered

Or We'd like to know about the breeding status of your pets. Which of the following statements about... = Some of my pets are spayed or neutered

Q10 Where did you get your pet spayed or neutered?

- Veterinarian’s office or clinic (1)
- Free or reduced cost clinic (2)
- Pet was already spayed/neutered when I got the pet (3)
- Unsure (4)
- Other (5) __________________________________________________

Q11 Have you ever had to give away one of your pets (e.g., to another person or animal shelter)?

- Yes (1)
- No (2)

Display This Question:

If Have you ever had to give away one of your pets (e.g., to another person or animal shelter)? = Yes

Q12 What were the reasons you had to give away your pet? Select all that apply.

- Moved to a location that didn’t allow pets (1)
- Pet medical issues (2)
- Costs of veterinary care (3)
- Cost of pet food (4)
- Other costs of owning a pet (5)
- Didn’t have time due to change in job/family situation (6)
- Pet behavioral issues (7)
- Allergies (8)
- Experienced housing instability (9)
- Landlord or insurance no longer allow my pet (11)
- Other (10) __________________________________________________

Display This Question:

If Have you ever had to give away one of your pets (e.g., to another person or animal shelter)? = Yes

Q13 Who have you given your pet or pets away to? [check all that apply for all pets given away]

- Animal shelter (1)
- Animal rescue group (2)
- Friend or family member (3)
- Other: (4) __________________________________________________

Q14 Which of the following best describes how you view your pet? [check all that apply]

- Family member (1)
- Companion/friend (2)
- Guard animal (3)
- Working animal (4)
- Service animal (5)
- Emotional support (6)
- Other: (7) __________________________________________________

End of Block: Basic pet history

Start of Block: Feed/Grooming/Pet Insurance

Q15 In the past two years, have you used a grooming service (e.g., hair cut, nail trim, etc) for your pet?

- Yes (1)
- No (2)

Display This Question:

If In the past two years, have you used a grooming service (e.g., hair cut, nail trim, etc) for your... = No

Q16 Why have you not used a grooming service for your pet? [check all that apply]

- My pet doesn’t need it (e.g., dog’s hair is short) (1)
- Don’t know where to get grooming (2)
- There isn’t a groomer nearby (3)
- Too expensive (4)
- I do my pet’s grooming myself (5)
- Other: (6) __________________________________________________

Q17 In the past two years, have you had trouble getting food for your pet?

- Yes (1)
- No (2)

Display This Question:

If In the past two years, have you had trouble getting food for your pet? = Yes

Q18 Why did you have trouble getting food? [check all that apply]

- Food is too expensive (1)
- Don’t know where to purchase food (2)
- Can't get transportation to purchase food (3)
- Difficulty finding/affording prescription or quality food (4)
- Other (5) __________________________________________________

Q19 Do you currently have pet insurance?

- Yes (1)
- No (2)

Display This Question:

If Do you currently have pet insurance? = No

Q20 What are some reasons why you don’t have pet insurance? (Check all that apply)

- Haven’t heard about it or don’t know enough (1)
- Can’t afford monthly costs (2)
- Don’t know how to get it (3)
- Don’t think its worth the monthly costs (4)
- Too difficult to sign up (5)
- Too difficult or confusing to file claims (6)
- Insurance doesn’t cover enough different types of procedures (7)
- Other: (8) __________________________________________________

End of Block: Feed/Grooming/Pet Insurance

Start of Block: Current/Past Veterinary Care

Q21 Have you ever taken your pet to see a veterinarian for any reason?

- Yes (1)
- No (2)

Display This Question:

If Have you ever taken your pet to see a veterinarian for any reason? = No

Q22 What are some reasons why you have not seen a veterinarian? [check all that apply]

- Too expensive (1)
- Don’t know of veterinarians near me (2)
- Can’t get to a veterinarian (3)
- Don’t trust veterinarians to provide care (4)
- I provide my own care (5)
- Other: (6) __________________________________________________

Q23 How do you decide when and if to get veterinary care for your pet? [check all that apply]

- Advice from friends/family (1)
- Veternarian's recommendation (5)
- Shelter/rescue recommendation (6)
- Online web search (2)
- Get care when pet is sick or hurt (3)
- Other (4) __________________________________________________

Display This Question:

If Have you ever taken your pet to see a veterinarian for any reason? = Yes

Q24 How did you choose which veterinarian to go to? [check all that apply]

- Closest veterinarian (1)
- Cost of veterinarian (2)
- Appointments available (3)
- Reviews (4)
- Recommendation from friend/family (5)
- Other: (6) __________________________________________________

Display This Question:

If Have you ever taken your pet to see a veterinarian for any reason? = Yes

Q25 How often do you typically go to the veterinarian?

- Every 6 months or more often (1)
- Yearly (2)
- Every two years (3)
- Only when my pet is sick (4)
- Have been just once (5)

| Page Break |  |
| --- | --- |

Display This Question:

If Have you ever taken your pet to see a veterinarian for any reason? = Yes

Q26 What services have you visited a veterinarian for? [check all that apply]

- Annual checkup (1)
- Heartworm, flea, or tick prevention (2)
- Vaccinations or shots (3)
- Spay/neuter (4)
- Needed medications (5)
- Dental care (6)
- Emergency (7)
- Treatment for a non-emergency illness, physical injury, or behavioral problem (8)
- Other (9) __________________________________________________

Display This Question:

If Have you ever taken your pet to see a veterinarian for any reason? = Yes

Q27 What type of veterinarian have you gone to? [Check all that apply] If you are unsure, make your best guess.

- Veterinary clinic or hospital (1)
- Mobile facility or van (2)
- Animal shelter or humane society (3)
- Community event with veterinarian (4)
- Pet superstore or pet store (6)
- Telemedicine (phone, video chat) (7)
- Other (8) __________________________________________________

Display This Question:

If Have you ever taken your pet to see a veterinarian for any reason? = Yes

Q28 Do you believe it is harder, easier, or about the same to find an appointment to see a veterinarian compared to 3 years ago?

- Much harder (1)
- Slightly harder (2)
- About the same (3)
- Slightly easier (4)
- Much easier (5)
- Unsure (6)

End of Block: Current/Past Veterinary Care

Start of Block: Barriers to Care

Q29 Has there been a time in the last two years where you tried to see a veterinarian but have been unable to?

- Yes (1)
- No (2)

Display This Question:

If Has there been a time in the last two years where you tried to see a veterinarian but have been u... = Yes

Q30 When you tried to see a veterinarian but were unable to, what type of care did you want to see a veterinarian for? [Check all that apply]

- Annual checkup (1)
- Heartworm, flea, or tick prevention (2)
- Vaccinations or shots (3)
- Grooming or nail trim (10)
- Spay/neuter (4)
- Needed medications (5)
- Dental care (6)
- Emergency care (7)
- Treatment for a non-emergency illness, physical injury, or behavioral problem (8)
- Other: (9) __________________________________________________

Display This Question:

If Has there been a time in the last two years where you tried to see a veterinarian but have been u... = Yes

Q31 When you tried to see a veterinarian but were unable to, what were the reasons that you were not able to get the care you wanted? [Check all that apply]

- I could not afford it (1)
- I did not have a car or other way to get my pet and myself to the clinic (2)
- The clinic was not open at a time I could come in (3)
- I could not find a veterinarian or care provider who speaks my language (4)
- I did not think a veterinarian could properly provide the care I needed (5)
- I was concerned a veterinarian or care provider would think badly of me for not seeking care sooner (6)
- I did not know where to get the care I wanted/needed for my pet (7)
- I did not have a leash or pet carrier to transport my pet (8)
- There is no clinic that is close enough to me (9)
- There were no available appointments at my nearby clinic (10)
- Other: (11) __________________________________________________

Q34 Please feel free to share here more information about any difficulties you have experienced trying to see a veterinarian for your pet.

________________________________________________________________

| Page Break |  |
| --- | --- |

Q32 How much do you think the services below would be helpful ***to you or others in your community*** facing problems getting the care they want or need for their pet?

|  | Not at all helpful (1) | Somewhat helpful (2) | Moderately helpful (3) | Very helpful (4) | Extremely helpful (5) |
| --- | --- | --- | --- | --- | --- |
| More mobile (traveling)/pop-up veterinary clinics providing preventative care services (e.g., vaccines or shots, dental care) and spay/neuter services in your community on certain days of the month (1) |  |  |  |  |  |
| A new low-cost clinic in your community that can provide **preventative care services** for pet owners (2) |  |  |  |  |  |
| A new low-cost clinic in your community that can provide **sick and emergency care** for pet owners (3) |  |  |  |  |  |
| Allowing pet owners to bring pets on public transportation for a scheduled or emergency veterinary appointment (4) |  |  |  |  |  |
| Ride-shares (e.g, uber, lyft) allowing pets in cars with their owners when going to a veterinary appointment (5) |  |  |  |  |  |
| A program that provides income-qualified pet owners with vouchers to receive a discount on veterinary services at a nearby clinic. (6) |  |  |  |  |  |
| A program for income-qualified pet owners that pays 80% of the costs of veterinary visits, and pet owners pay only 20% (7) |  |  |  |  |  |
| Easy access to information on and help with signing up for pet insurance (8) |  |  |  |  |  |
| Funds that you can apply for to cover a portion of the costs of your veterinary visit (9) |  |  |  |  |  |
| Affordable pet health insurance options (10) |  |  |  |  |  |
| Vaccine clinics at your local animal shelter (11) |  |  |  |  |  |
| Information on where to access veterinarians that speak languages other than English (12) |  |  |  |  |  |
| More availability of pet food pantries in your community (13) |  |  |  |  |  |
| Vouchers for income-qualifying pet owners to purchase quality pet food at a discounted price (14) |  |  |  |  |  |
| Guidance on where to shop for low cost pet foods and free pet food delivery (15) |  |  |  |  |  |
| Availability of telemedicine options, where you can meet with a veterinarian on a phone/video rather than going to a clinic in person (16) |  |  |  |  |  |
| More availability of veterinary appointments in general (17) |  |  |  |  |  |
| More availability of veterinary appointments outside of traditional working hours (8-5, Monday through Friday) (18) |  |  |  |  |  |

Q55 How much do you think the services below would be helpful ***to you or others in your community*** facing problems getting the care they want or need for their pet?

|  | Not at all helpful (1) | Somewhat helpful (2) | Moderately helpful (3) | Very helpful (4) | Extremely helpful (5) |
| --- | --- | --- | --- | --- | --- |
| More mobile (traveling)/pop-up veterinary clinics providing preventative care services (e.g., vaccines or shots, dental care) and spay/neuter services in your community on certain days of the month (1) |  |  |  |  |  |
| A new low-cost clinic in your community that can provide **preventative care services** for pet owners (2) |  |  |  |  |  |
| A new low-cost clinic in your community that can provide **sick and emergency care** for pet owners (3) |  |  |  |  |  |
| Allowing pet owners to bring pets on public transportation for a scheduled or emergency veterinary appointment (4) |  |  |  |  |  |
| Ride-shares (e.g, uber, lyft) allowing pets in cars with their owners when going to a veterinary appointment (5) |  |  |  |  |  |
| A program that provides income-qualified pet owners with vouchers to receive a discount on veterinary services at a nearby clinic. (6) |  |  |  |  |  |
| A program for income-qualified pet owners that pays 80% of the costs of veterinary visits, and pet owners pay only 20% (7) |  |  |  |  |  |
| Easy access to information on and help with signing up for pet insurance (8) |  |  |  |  |  |
| Funds that you can apply for to cover a portion of the costs of your veterinary visit (9) |  |  |  |  |  |
| Affordable pet health insurance options (10) |  |  |  |  |  |
| Vaccine clinics at your local animal shelter (11) |  |  |  |  |  |
| Information on where to access veterinarians that speak languages other than English (12) |  |  |  |  |  |
| More availability of pet food pantries in your community (13) |  |  |  |  |  |
| Vouchers for income-qualifying pet owners to purchase quality pet food at a discounted price (14) |  |  |  |  |  |
| Guidance on where to shop for low cost pet foods and free pet food delivery (15) |  |  |  |  |  |
| Availability of telemedicine options, where you can meet with a veterinarian on a phone/video rather than going to a clinic in person (16) |  |  |  |  |  |
| More availability of veterinary appointments in general (17) |  |  |  |  |  |
| More availability of veterinary appointments outside of traditional working hours (8-5, Monday through Friday) (18) |  |  |  |  |  |

Q33 What other resources or programs could help you and/or people in your community be able to get the veterinary care you/they want or need for your/their pet?

________________________________________________________________

________________________________________________________________

________________________________________________________________

________________________________________________________________

________________________________________________________________

End of Block: Barriers to Care

Start of Block: VTS/VPA

Q41 The next few questions ask about your opinion on a few ideas that have been suggested as solutions for increasing the availability of veterinary care to more pets. People may have different opinions on these ideas, so we’d like to know what you think about each.

 One possible/suggested solution for increasing access to veterinary services when they are wanted is to add different kinds of veterinary professionals. These professionals would be trained to provide some of the services that veterinarians usually provide while under the supervision of a veterinarian. These include: Registered Veterinary Technicians (RVTs), Veterinary Technicians Specialist (VTSs), and Veterinary Professional Associates (VPAs).

  **Registered Veterinary Technicians (RVTs)** are similar to a registered nurse (RN) in human healthcare. To become a registered veterinary technician, most people graduate from a 2 or 4-year veterinary technology program, are trained in the care and handling of animals, their normal and abnormal life processes, medical and surgical nursing, anesthesiology, diagnostic imaging, and clinical laboratory procedures, pass a minimum competency exam, are licensed by the state of Colorado, and work under the supervision of a veterinarian.

 **Veterinary Technician Specialists (VTSs)** are registered veterinary technicians who have additional training in a specified field of veterinary medicine (e.g., dermatology, dental care) and pass an additional competency exam. They are similar to a nurse practitioner in human healthcare.

 **Veterinary Professional Associates (VPA)** are a new type of veterinary professional that some have proposed creating. VPAs would be similar to a physician’s assistant (PA) in human healthcare and would graduate from a Masters of Veterinary Clinical Care degree program, would be trained in clinical case management, and would work under the supervision of a veterinarian.

  Some advocate for these professionals to be able to provide some of the services that veterinarians usually provide. They say that having these professionals provide services instead of veterinarians can lead to lower cost of care and the ability for clients to get into appointments faster. Others say that these professionals should not be able to provide some of the services that veterinarians usually provide. They say that these professionals won't have the same expertise as veterinarians so they will not be able to provide care in complex situations.

  As a pet owner, we would like to know what services, if any, you would feel comfortable having these professionals provide for your pet instead of a veterinarian. For each, check if you would feel comfortable only having a veterinarian provide the service or if you would feel comfortable having a Registered Veterinary Technician, a Veterinary Technology Specialist, or a Veterinary Professional Associates perform each [check “Veterinarian only,” “RVT,” “VTS,” or “VPA”, or “Not sure” for each below]

|  | Registered Veterinary Technician (RVT) (2) | Veterinary Technician Specialists (VTS) (3) | Veterinary Professional Associate (VPA) (4) | Only Veterinarian (DVM) (5) | Not Sure (6) |
| --- | --- | --- | --- | --- | --- |
| Annual exam/check-up (1) |  |  |  |  |  |
| Vaccine administration (2) |  |  |  |  |  |
| Spaying (3) |  |  |  |  |  |
| Neutering (4) |  |  |  |  |  |
| Dental care (e.g., cleaning dirty teeth) (5) |  |  |  |  |  |
| Dental surgery (e.g., removing teeth) (6) |  |  |  |  |  |
| Treatment of non-urgent sickness/medical conditions (e.g., skin rashes, ear infections, lameness) (7) |  |  |  |  |  |
| Treatment of urgent sickness/medical conditions (vomiting, not eating) (8) |  |  |  |  |  |

Q42 What information would help you decide whether you would feel comfortable having a VPA, RVT, or VTS provide services for your pet instead of a veterinarian? [Check all that apply]

- Knowing what classes they take (1)
- Knowing their hands-on technical trainings (2)
- Cost difference in getting treatment from these professionals instead of a veterinarian (3)
- Knowing that the professional is licensed (4)
- Other: (5) __________________________________________________

End of Block: VTS/VPA

Start of Block: Telehealth

Q35 To increase the number of pets that veterinary professionals can care for, some have suggested increasing the use of telemedicine. Telemedicine is the practice of medicine using technology (like a phone or computer) to provide appointments at a distance. A telemedicine appointment would involve a video chat with a veterinarian. Would you feel comfortable seeing a veterinarian through a telemedicine appointment for your pet?

- Yes (1)
- No (2)

Display This Question:

If To increase the number of pets that veterinary professionals can care for, some have suggested in... = No

Q36 Why would you NOT feel comfortable seeing a veterinarian through a telemedicine appointment? [Check all that apply]

- I don’t have the technology to access it (1)
- The technology seems difficult to use (2)
- Concerned about the quality of care versus an in person appointment (3)
- I don’t know if the care my pet might need can be done with telemedicine (4)
- Other: (5) __________________________________________________

Display This Question:

If To increase the number of pets that veterinary professionals can care for, some have suggested in... = Yes

Q37 Would you feel comfortable seeing a veterinarian for the first time via a virtual telemedicine visit?

- Yes (1)
- No (2)

Q38 What information would help you feel more comfortable seeing a veterinarian through a telemedicine appointment for your pet? [Check all that apply]

- A list of services that are able to be done through telemedicine versus services that can only be done in person (1)
- Cost of a telemedicine appointment versus an in person appointment (2)
- A guide on what is needed and how to use the technology for a telemedicine appointment (3)
- Other (4) __________________________________________________

Q39 Please select the effect that access to telemedicine would have on whether you contact a veterinarian about concerns you have about your pet(s).

- Greatly increase likelihood of contacting veterinarian (1)
- Somewhat increase likelihood of contacting veterinarian (2)
- No impact on whether I contact a veterinarian (3)
- Somewhat decrease likelihood of contacting veterinarian (4)
- Greatly decrease likelihood of contacting veterinarian (5)

End of Block: Telehealth
